# MRTF-A gain-of-function in mice impairs homeostatic renewal of the intestinal epithelium

**DOI:** 10.1038/s41419-023-06158-4

**Published:** 2023-09-28

**Authors:** Anurag Kumar Singh, Amrita Rai, Anja Weber, Martin Gericke, Klaus-Peter Janssen, Markus Moser, Guido Posern

**Affiliations:** 1 Institute for Physiological Chemistry, Medical Faculty, Martin Luther University Halle-Wittenberg, 06114 Halle (Saale), Germany; 2 Department of Structural Biochemistry, Max Planck Institute of Molecular Physiology, 44227 Dortmund, Germany; 3 Institute of Anatomy and Cell Biology, Medical Faculty, Martin Luther University Halle-Wittenberg, 06120 Halle (Saale), Germany; 4 Institute of Anatomy, Medical Faculty, Leipzig University, 04103 Leipzig, Germany; 5 Department of Surgery, Klinikum rechts der Isar, Technical University Munich, 81675 Munich, Germany; 6 Department of Molecular Medicine, Max Planck Institute of Biochemistry, 82152 Martinsried, Germany; 7 Institute of Experimental Hematology, School of Medicine, Technical University Munich, 81675 Munich, Germany

**Keywords:** Transcription factors, Mouse

## Abstract

The actin-regulated transcription factor MRTF-A represents a central relay in mechanotransduction and controls a subset of SRF-dependent target genes. However, gain-of-function studies in vivo are lacking. Here we characterize a conditional MRTF-A transgenic mouse model. While MRTF-A gain-of-function impaired embryonic development, induced expression of constitutively active MRTF-A provoked rapid hepatocyte ballooning and liver failure in adult mice. Specific expression in the intestinal epithelium caused an erosive architectural distortion, villus blunting, cryptal hyperplasia and colonic inflammation, resulting in transient weight loss. Organoids from transgenic mice repeatedly induced in vitro showed impaired self-renewal and defective cryptal compartments. Mechanistically, MRTF-A gain-of-function decreased proliferation and increased apoptosis, but did not induce fibrosis. MRTF-A targets including *Acta2* and *Pai-1* were induced, whereas markers of stem cells and differentiated cells were reduced. Our results suggest that activated MRTF-A in the intestinal epithelium shifts the balance between proliferation, differentiation and apoptosis.

## Introduction

Myocardin-related transcription factor A (MRTF-A/MAL/MKL1) belongs to the myocardin family of transcriptional coactivators controlling the expression of subsets of serum response factor (SRF) - dependent target genes [1–3]. MRTF-A and its close relative, MRTF-B, regulate several immediate early genes and cytoskeletal components, thereby controlling critical biological functions [4, 5]. By means of its N-terminal RPEL domain, MRTF-A binds to monomeric G-actin, thus rendering the protein transcriptionally inactive unless Rho-dependent actin dynamics result in MRTF release and activation of target gene expression [6–10].

MRTF/SRF-mediated transcriptional regulation is important for various tissues, as demonstrated by developmental defects in knockout animals. MRTF-A null mice harbor a defective myoepithelium in the mammary gland, whereas MRTF-A and -B double knockout is early embryonically lethal, presumably due to redundancy in MRTF-A and MRTF-B functions [11–14]. Similarly, severe gastrulation defects occur upon deletion of the Srf gene locus [15]. In breast and kidney epithelial cells, MRTF-A activation has been reported to upregulate SNAI2 and ACTA2 and induce an epithelial-mesenchymal or epithelial-myofibroblast transition (EMT/EMyT), which results in a profibrotic epithelial phenotype [16–21].

The intestinal epithelium balances two main functions, namely the selective nutrient uptake and the protective surface barrier. To reliably fulfill both tasks, the intestinal epithelium is rapidly renewed, with the entire epithelium turned over every 3–7 days [22]. Starting from Lgr5^+^ stem cells in the crypts, progenitor cells divide and differentiate into six functionally specialized cell types, including paneth cells as multifunctional stem cell guardians, mucin-producing goblet cells, and the bulk of absorptive enterocytes in the villi [23–25]. Pushed upwards, the short-lived cells are eventually shedded at the top of the villi and undergo anoikis in the intestinal lumen. Injury, viruses, toxins, stress and autoinflammation, as occurs in inflammatory bowel diseases (IBD), can severely damage the epithelium by compromising the sensitive cryptal stem cell compartment and cause villus atrophy [26]. Partial breakdown of the protective surface barrier exposes the intestine to the harmful gut content, thereby causing acute inflammation and further damaging the intestinal architecture. Rapid repair by epithelial self-renewal is essential to reestablish barrier integrity, either by utilizing reserve stem cells, or reacquisition of stemness by multiple cell types, or adaptive differentiation of villus epithelial cells [27–29].

During development, MRTF-A is profoundly expressed in epithelia, including epithelial cells of the small intestine and colon [30]. However, the role of MRTFs in the intestinal epithelium has not been investigated yet. In addition, MRTF gain-of-function studies in vivo are lacking. By ectopic expression in cell lines, we have previously observed that activated MRTF-A is anti-proliferative and pro-apoptotic, whilst it causes luminal filling, polarization defects and induction of EMT markers in 3D MCF10A cultures [20, 31, 32]. To investigate the effects of MRTF-A gain-of-function in vivo, we generated MRTF-A transgenic mice in which we partially deleted the regulatory RPEL domain. Expression of this constitutively active MRTF-A variant during embryogenesis was lethal, as was its activation in the adult due to rapid liver failure. Induced expression of gain-of-function MRTF-A in the intestinal epithelium resulted in weight loss, villus atrophy, cryptal hyperplasia and inflammation, whilst intestinal shortening was not observable. MRTF-A target genes were induced, whereas most markers of intestinal cell types, including the Lgr5^+^ stem cells, were downregulated. Organoid cultures from transgenic mice showed that the epithelial atrophy was caused by increased apoptosis, decreased proliferation and reduced crypt functionality. The recovery of the mice in the second week could be recapitulated in organoids and suggested that cells escaping the Cre-mediated recombination fueled the epithelial self-renewal.

## Materials and methods

### Generation of the inducible MRTF-A ∆ 3–5 (Rosa26:MRTF-A) mice

The HA-tagged MRTF-A cDNA lacking the RPEL1/2 domains and the intervening spacer1 (named MRTF-A Δ3–5) was cloned into an established Rosa26 targeting vector, replacing the Cas9-P2AEGFP ORF (Addgene #61408) [33]. To allow conditional expression, this vector contains a STOP cassette flanked by loxP sites between a CAG promoter and the cDNA. After electroporation into R1 mouse embryonic stem cells (ATCC), a clone positively tested for successful homologous recombination was injected into blastocysts to generate chimeric mice. The germ line transmission of chimeras was validated by backcrossing with C57BL/6n mice (Charles River Laboratories). Rosa26:MRTF-A^*wt/fl*^ were interbred to validate mendelian segregation and fertility of male and female mice. Global activation of MRTF-A was achieved by breeding with heterozygous Nestin-Cre [34]. Homozygous transgenic mice (Rosa26:MRTF-A^*fl/fl*^) were mated with heterozygous Rosa26:CreERT2 [35], Villin-Cre or Villin-CreERT2 [36] mice to generate offspring of constitutional, inducible or conditional MRTF-A gain-of-function mice.

For induction, 10 mg/ml tamoxifen (TRC-T006000-25G, BIOZOL) was prepared freshly in MIGLYOL 812 (CSLO3274.250, VWR) by shaking it at 37 °C overnight in the dark. During experiments, it was stored at 4 °C for a maximum of one week and prewarmed to 37 °C before intraperitoneal injection of 100 µl to delete the STOP cassette as indicated. All mouse work was approved by the local authorities (Az. 42502-2-1592 MLU), and mice were maintained under the guidelines of the animal facility of the Martin Luther University of Halle-Wittenberg.

### Genotyping

Polymerase chain reaction (PCR) was used for validation of homologous recombination of the targeting construct. All the primers used are mentioned in Supplementary Table S1. For the confirmation of the intended locus, primer pairs were designed beyond the homologous arms on both sides of the Rosa26 locus and used with another primer pair within the targeting construct. An internal primer pair was used for confirming the entirety of the targeting construct and detecting the STOP cassette between the CAG promoter and the cDNA.

Genotyping was performed from ear excisions or organoids. After lysis in 400 µl lysis buffer (100 mM Tris-HCl, 5 mM EDTA, 0.2% SDS, 200 mM NaCl, pH 8–8.5) supplemented with 2 µl Proteinase K (New England Biolabs), DNA was precipitated with isopropanol. DNA concentration was determined using NanoDrop ND-1000 (NanoDrop) and 150 ng were used for subsequent PCR analysis using specific primers for the detection of Cre or the floxed STOP cassette. The PCR products were separated on a 1% agarose gel and imaged using the Gel stick system (Intas Science Imaging).

### Luciferase reporter assay

Reporter assays were described previously [8]. Briefly, NIH3T3 or HCT-8 cells were seeded in a 12-well plate and transfected with 250 ng of the p3D.A-Luc, 50 ng pRL-TK luciferase reporter plasmids and 700 ng of the indicated MRTF-A constructs, using polyethylenimine (23966-1, Polysciences). Transfected cells were cultured under 0.5% serum conditions for 24 h. To assess the Rosa26 targeting construct, HCT-8 cells stably selected with G418 were treated with TAT-Cre Recombinase (SCR508, Merck). Firefly luciferase activity was measured by Dual-Glo Luciferase Assay (Promega) and normalized to the renilla luciferase and the vector control.

### Histological analysis

H&E (Art. No. 9194.2, Carl Roth, Karlsruhe, DE), PAS (Art. No. HP01.1, Carl Roth) and Sirius Red (13422.01000, Morphisto, Offenbach, DE) staining was performed according to the manufacturers’ instructions. Pictures were taken by EVOS™ XL Core Imaging System (Thermo Fisher Scientific, Dreieich, DE) or Axioimager equipped with Axiocam MRc (Zeiss, Jena, DE). PAS and Sirius Red staining was quantified using ImageJ software (NIH, Bethesda, MD, USA, http://rsb.info.nih.gov/ij/). The positively stained area is represented as percentage of the total tissue area of the section.

For immunofluorescence staining, the mouse intestine was rinsed with ice-cold phosphate buffer saline (PBS) and fixed for 2 h at 4 °C with 2% paraformaldehyde in PBS. Fixed tissue was rinsed with PBS and transferred to 30% sucrose in PBS overnight. The tissue was embedded in tissue-freezing medium (TissueTek O.C.T., Sakura, JPN). Cryosectioning was done with a microtome cryostat at −20 °C and 10-μm-thick sections were collected on microscope slides (SuperFrost Plus, Menzel Gläser, DE). Sections were boiled in EDTA antigen retrieval buffer (1 mM EDTA, 10 mM Tris pH 9.0, 0.05% Tween) for 10 min. Staining was performed after blocking with background-reducing buffer (#S302283-2, Dako) for 20 min with anti-HA (1:500, H6908, Sigma, Taufkirchen, DE) antibodies for 2 h at room temperature. Staining of Krt20 (1:500, #13063, Cell Signaling), Chromogranin A (Chga, 1:500, ab15160, Abcam), lysozyme EC 3.2.1.17 (1:500, A009902-2, Agilent Dako) and Olfm4 (1:500, #39141, Cell Signaling) was performed accordingly but without antigen retrieval. Incubation with secondary antibody (Alexa Fluor 488-labeled goat anti rabbit IgG, 1:1000, Molecular Probes, Eugene, OR, USA) and DAPI was performed for 2 h at room temperature. Pictures were taken using a Axio Observer fluorescence microscope equipped with Plan Apochromat 20×/0.8 objective and Axiocam MRm (Zeiss).

### Primary organoid culture, staining, and swelling assays

Intestinal organoids were prepared from either MRTF-A^*fl/wt*^; Villin-CreERT2^−/−^ (M^*fl/wt*^; VC^−/−^, control) or MRTF-A^*fl/wt*^; Villin-CreERT2^+/−^ (M^*fl/wt*^; VC^+/−^, experimental) mice and cultured in the IntestiCult™ Organoid Growth Medium (Mouse) (Cat. No. 06005, Stemcell Technologies, Vancouver, Canada) according to the manufacturer’s instructions. Recombination in organoids was induced with 0.15 µM tamoxifen (1 mM stock in DMSO) at the time points indicated. Immunofluorescence staining was done in ibidi µ-Slide 8 well (Cat. No. 80826, ibidi, Gräfelfing, DE) as described [37]. For Mucin2, organoids were boiled in citrate antigen retrieval buffer (10 mM Sodium citrate, 0.05% Tween 20, pH 6.0) for 30 min prior to incubation with Muc2 antibodies (1:500, GTX100664, GeneTex). Additional antibodies used were cleaved caspase-3 (1:500, #9664, Cell Signaling, Leiden, Netherlands), Ki-67 (1:500, #9129, Cell Signaling) and phospho-histone H3 (Ser10) (1:1000, #06-570, Merck, Darmstadt, DE). Organoids were stained with secondary antibody (1:1000, Alexa 488 goat anti rabbit IgG, Molecular Probes) together with DAPI. Images were taken by a Zeiss Axio Observer fluorescence microscope equipped with ApoTome2 and quantified using ImageJ software. The intensity of staining was normalized to the area and the DAPI staining.

For forskolin-induced swelling assays, tamoxifen (0.15 µM) treated organoids were grown for two days, splitted again into flat bottom 96-well plate in 5 µl matrigel containing 20-80 organoids and 100 µl culture medium and left overnight for recovery to ensure similar size organoids. After the addition of forskolin (10 µM) images were taken every 5 min for another 50 min, using 10 wells per condition. Individual organoid areas were quantified using ImageJ software, and swelling was quantified as the average increase of the initial area.

### Immunoblotting

The jejunum was first washed extensively with ice-cold PBS to remove all debris, then washed three time with 3 mM N-Acetyl-L-cysteine (A9165, Sigma) to remove the attached mucus. The cleaned jejunum was cut into small pieces of around 3 mm and incubated in chilled 30 mM EDTA for 20 min with agitation at room temperature. Villi and crypts were collected by pipetting the tissue up and down in PBS/0.1% BSA with a 5 ml pipette. All the intestinal cells from three repetitions were collected by centrifugation and were washed three times with 3 mM N-Acetyl-L-cysteine. Collected cells were lysed in RIPA buffer (0.1% SDS, 0.5% Desoxycholate, 1% Triton X-100, 1 mM EDTA, 150 mM NaCl, 50 mM Tris pH 7.4) containing protease and phosphatase inhibitors (1 mM PMSF, 10 mg/ml Aprotinin, 2 mM sodium orthovanadate) and protein concentration was estimated by BCA assay (Thermo Fisher) according to the manufacturer’s protocol. Tamoxifen-treated organoids were washed two times with ice-cold PBS to remove the matrigel prior to lysis. Immunoblotting was done against vinculin (V9131, Sigma, 1:1000), SRF (Cat. No. 61385, Active Motif, 1:1000), α-SMA (A5228, Sigma, 1:1000), Snai2 (MAB4271, Millipore, 1:500), cleaved caspase-3 (9664, Cell Signaling, 1:500), phospho-Histone H3 (Ser10) (53348T, Cell Signaling, 1:500) Tubulin (T9026, Sigma, 1:3000) and HA (H6908, Sigma, 1:1000) overnight at 4 °C. Fluorophore-conjugated secondary antibodies IRDye 700 or IRDye 800 (1:15000, LICOR Biosciences) or HRP-conjugated secondary antibodies (1:5000, Cell Signalling) were incubated for 1 h at room temperature. Imaging and quantifications were done using either the Odyssey Image Scanner System with the software Image Studio V 3.1.4 (LI-COR Biosciences, Cambridge, UK), or the ChemiDoc MP imaging system (Biorad), followed by quantification using ImageJ software, as described before [38].

### Expression analysis via qRT-PCR

RNA was extracted either from intestinal segments or intestinal organoids using NucleoSpin RNA Mini kit for RNA purification (Macherey-Nagel) according to the manufacturer’s instructions. For cDNA synthesis, the Verso cDNA Synthesis kit (Thermo Scientific) was used with 1 μg of total RNA and Oligo-dT primers. Transcript analysis was performed using the LightCycler 480 System (Roche, Mannheim Germany) and DyNamo Colorflash SYBR Green qPCR kit (Thermo Scientific) according to the manufacturer’s instructions using specific primers for the indicated genes. Primer sequences used in this study are presented in the supplementary table S1. Calculations were done using the ΔΔ cycle threshold (Ct) method [39].

### Statistical analysis

Data are presented as mean ± SEM. Statistical analysis was performed using unpaired Student’s *t*-test, where *p* < 0.05 was considered statistically significant, and p values are denoted as follows: *p < 0.05, **p < 0.01 and ***p < 0.001.

## Results

### Characterization of MRTF-A Δ3–5 as a gain-of-function variant

**Fig. 1:**
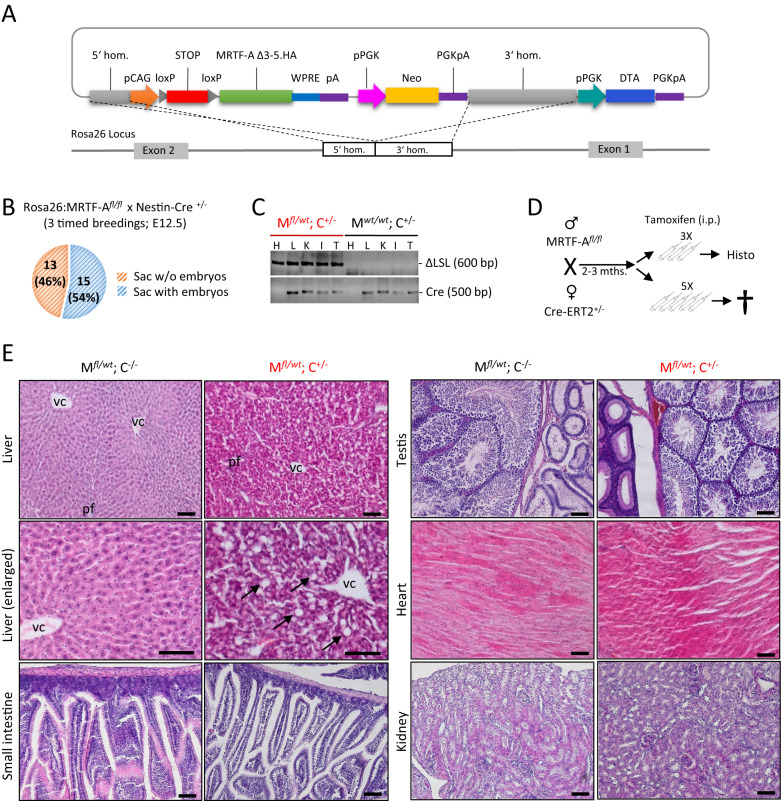
Generation of a conditional transgenic mouse line harboring a MRTF-A gain-of-function. **A** Schematic representation of the *Rosa26* targeting vector to generate the constitutive active MRTF-A. The expression of the MRTF-A Δ3–5 cDNA under the CAG promoter is prevented by a loxP-STOP-loxP cassette, which can be deleted by a constitutive, inducible or tissue specific Cre-recombinase expression. **B** Embryonic lethality upon interbreeding of homozygous floxed mice with constitutive Nestin-Cre. Embryonic sacs were analyzed at E12.5. **C** Genotyping of tamoxifen-injected mice harboring a CreERT2 transgene. Shown is the deletion of the STOP cassette (ΔLSL) and the Cre-ERT2 transgene in tissues of double-positive mice (M^*fl/wt*^ C^+/−^) compared to control mice (M^*wt/wt*^ C^+/−^), shown by PCR genotyping. H, heart; L, liver; K, kidney; I, intestine; T, testis. **D** Breeding and injection scheme for inducible, ubiquitous expression in adult mice. Three daily tamoxifen injections were followed by histological analysis at day 4, whereas no double-positive mice survived 5 consecutive injections. **E** H&E staining of organs prepared from tamoxifen-injected mice as depicted in (**D**). Arrows indicate ballooning and vacuolization of the hepatocytes, resulting in liver failure and subsequent lethality in double-positive mice. vc vena centralis, pf portal field. Scale bars, 100 μm.

The analysis of the physiological functions of MRTF-A has been impeded by the functional redundancy between MRTF-A and MRTF-B, by the early embryonic lethality of double-knockouts, and by the lack of gain-of-function studies in vivo. We therefore generated transgenic mice allowing conditional MRTF-A gain-of-function studies. A loxP-STOP-loxP vector was created to permit Cre-induced recombination and subsequent MRTF-A expression from the *Rosa26* locus. To design a constitutively active MRTF-A, we cloned MRTF-A Δ3–5 which lacks the sequences from exon 3 to exon 5. This construct encodes a protein with an in-frame deletion of RPEL motif one, spacer one and RPEL motif two, which are part of the negative regulatory N-terminal region of MRTF-A (Fig. 1A). We chose this Δ3–5 construct to reduce the known anti-proliferative effects of MRTF-A lacking the entire N-terminus (MRTF-A ΔN) [31]. Moreover, MRTF-A Δ3–5 maintains important phosphorylation sites on the N-terminus which are involved in nuclear export, thus theoretically permitting some regulation by cellular cues [40].

Constitutive activity of this construct was firstly tested in the well-characterized NIH3T3 cell culture model. Transiently transfected MRTF-A Δ3–5 strongly induced a MRTF/SRF-dependent luciferase reporter, but less than the previously characterized MRTF-A ΔN. Consistently, whilst MRTF-A ΔN accumulated in the nucleus, MRTF-A Δ3–5 localized to both the nucleus and the cytoplasm (Supplementary Fig. S1A, B). Stable transfection of colorectal HCT-8 cells with the conditional targeting vector revealed an inducible expression of HA-tagged MRTF-A Δ3–5 upon treatment with recombinant HTNC-Cre recombinase in vitro, accompanied by significant reporter induction (Supplementary Fig. S1C, D).

Following electroporation in R1 (129 Sv) embryonic stem cells, neomycin-resistant colonies were validated by PCR for homologous recombination (Supplementary Fig. S2A). One of the selected ESC clones was injected into blastocysts to generate germ line chimeras, and after backcrossing the F1 generation was characterized by genotyping (Supplementary Fig. S2B). Heterozygous founder mice harboring the floxed MRTF-A Δ3–5 (hereafter named *Rosa26*:MRTF-A^*fl/wt*^) were then intercrossed. Mice homozygous for *Rosa26*:MRTF-A showed in quantitative PCR that neomycin phosphotransferase DNA content is comparable to that of Gapdh, but twice as high as in heterozygotes, indicating single integration of the construct (Supplementary Fig. S2C).

### Ubiquitous expression of activated MRTF-A in transgenic mice

Breeding of *Rosa26*:MRTF-A^*fl/wt*^ with a Cre deleter mouse strain [34] resulted in non-mendelian ratios, in contrast to *Rosa26*:MRTF-A^*fl/wt*^ intercrosses (Table 1). By genotyping, we could not detect any double-positive mice with the corresponding deletion of the STOP cassette, whilst the remaining genotypes were equally distributed. Preliminary characterization of prenatal embryos in timed breeding of homozygous MRTF-A transgenes with heterozygous Cre mice revealed the absence of embryos in 46% of the uterine sacs at day E12.5 (Fig. 1B), presumably resulting from embryonic maldevelopment and reabsorption. This strongly suggests an early embryonic lethality of mice expressing the constitutively active MRTF-A Δ3–5 ubiquitously. Due to the early stage, we were as yet unable to further characterize the plausible cause of death of these embryos.

**Table 1:** Outcome of mouse breedings.

Rosa26:MRTF-A^*fl/wt*^ crossed with			
	Number of breeding	Viable offsprings	Offspring genotype (expected %); actual %
Rosa26:MRTF-A^*fl/wt*^	16	128	31 fl/fl (25%); 24.2%
			64 fl/wt (50%); 50.0%
			33 wt/wt (25%); 25.8%
Nestin-Cre^+/−^	8	29	0 ∆/wt; +/− (25%); 0%^a^
			9 wt/wt; +/− (25%); 32.1%
			9 fl/wt; −/− (25%); 32.1%
			11 wt/wt; −/− (25 %); 39.3%
Rosa26:MRTF-A^*fl/fl*^ crossed with			
Rosa26:CreERT2^+/−^	5	39	22 fl/wt; +/− (50%); 56.4%
			17 fl/wt; −/− (50%); 43.6%
Villin-Cre^+/−^	4	11	0 ∆/wt; +/− (50%); 0 %^a^
			11 fl/wt; −/− (50%); 100%
Villin-CreERT2^+/−^	11	86	40 fl/wt; +/− (50%); 46.5%
			46 fl/wt; −/− (50%); 53.5%

^a^Embryonic lethality.

To enable expression of MRTF-A Δ3–5 which is postnatally inducible by tamoxifen, we bred homozygous *Rosa26*:MRTF-A^*fl/fl*^ mice with Cre-ERT2 transgenics [35]. These breedings showed normal litter sizes and the expected 50% mendelian ratio of the offspring upon genotyping (Table 1). Upon tamoxifen injection, deletion of the lox-STOP-lox cassette was confirmed by PCR in all organs tested, including heart, liver, kidney, intestine and testis (Fig. 1A, C). We then intraperitoneally injected two to three months old double-positive and control mice lacking CreERT2 for three consecutive days with 1 mg of tamoxifen, and sacrificed them at day 4 for further analysis (Fig. 1D).

Strikingly, organ analysis showed that tamoxifen-injected *Rosa26*:MRTF-A^*fl/wt*^ Cre-ERT2^+/−^ mice exhibited visibly diseased livers and cholestasis. Histological analysis showed massive ballooning of the hepatocytes with an accumulation of lipids and signs of necrosis, despite a generally maintained liver architecture and little immune infiltration (Fig. 1E). At this stage, all other organs analyzed seemed to be little affected, suggesting that this rapid liver response is causal for lethality in adult mice ubiquitously overexpressing MRTF-A Δ3–5 (Fig. 1E and Supplementary Fig. S3). However, the extraordinary speed and strength of this lethal phenotype prevented a more detailed investigation of the unknown etiology.

### Conditional activation of MRTF-A in the intestinal epithelium

**Fig. 2:**
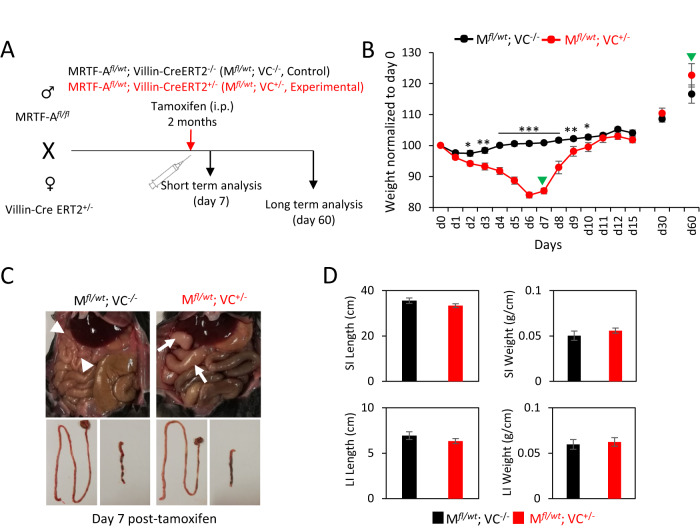
Induced gut-specific MRTF-A gain-of-function results in rapid and transient weight loss. **A** Schematic of the breeding, injection and analysis of MRTF-A^*fl/wt*^ Villin-CreERT2^+/−^ -mice. **B** Normalized mouse weight over time. *Green arrowheads* indicate sampling for short and long term analysis. **C** Macroscopic image of the intestine after 7 days. *White arrowheads*, normal intestine, *white arrows*, inflamed intestine. **D** Length and relative weight of the small intestine (SI) and the large intestine (LI) after 7 days. Error bars, SEM (*n* = 3). *p ≤ 0.05, **p ≤ 0.01, ***p ≤ 0.001.

To further analyze the functional effects of our MRTF-A gain-of-function mice, we focussed on the intestinal epithelium. Our first approach to interbred Villin-Cre transgenics with our floxed MRTF mice did not generate viable offspring, again indicating embryonic lethality (Table 1). Therefore we crossed homozygous *Rosa26*:MRTF-A^*fl/fl*^ mice with Villin-CreERT2 mice, allowing conditional and tamoxifen-inducible MRTF-A gain-of-function in intestinal epithelial cells [36]. Double-positive mice (*Rosa26*:MRTF-A^*fl/wt*^ Villin-Cre-ERT2^+/−^), injected at 2 months of age with a single dose of tamoxifen, lost more than 15% body weight within 6–7 days, but fully recovered until day 11 (Fig. 2A, B). Obvious signs of diarrhea were not observed. Upon dissection at day 7, slightly inflamed intestinal areas were visible in double-positive mice, suggesting a transient intestinal malfunction and/or inflammation (Fig. 2C). However, the dissected small and large intestines did not show obvious macroscopic defects nor altered length or weight at day 7 (Fig. 2C, D).

**Fig. 3:**
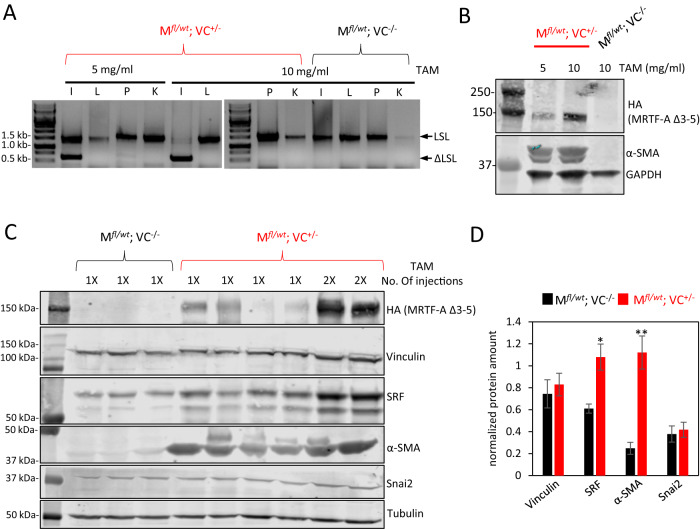
Tamoxifen injection of MRTF-A^*fl/wt*^ Villin-CreERT2^+/−^ -mice genetically activates MRTF-A Δ3–5 and upregulates target genes. **A** Genotyping of the organs of the double-positive (M^*fl/wt*^ VC^+/−^) and control (M^*fl/wt*^ VC^−/−^) mice at day 7 after injection with 100 µl of the indicated tamoxifen concentration. I, intestine; L, liver; P, pancreas; K, kidney. LSL indicates the PCR product containing the entire loxP-STOP-loxP cassette, ΔLSL the recombined amplicon with deleted STOP cassette. **B** Immunoblotting of intestinal protein extracts with anti-HA antibodies detecting constitutively active MRTF-A, anti-smooth muscle actin (α-SMA) and GAPDH as a control. **C** Immunoblot analysis of intestines from several mice with the indicated genotype, injected once or twice with 10 mg/ml tamoxifen. **D** Quantification of relative protein amounts in intestines of injected double-positive and control mice. Error bars, SEM (*n* = 3). *p ≤ 0.05, **p ≤ 0.01.

Organ-specific recombination was analyzed by genotyping of the intestine, liver, pancreas and kidney. The STOP cassette was not removed in the control mice and in the liver, pancreas and kidney of double-positive mice; only intestinal tissue showed evidence of a dose-dependent recombination at day 7 (Fig. 3A). Concomitantly, HA-tagged MRTF-A Δ3–5 protein and its known target smooth muscle actin were increasingly detected in intestinal protein extracts of double-positive mice, but were absent in controls, as expected (Fig. 3B). Further analysis of putative target proteins in several mice sacrificed at day 7 showed a correlation of MRTF-A Δ3–5 expression with SRF and smooth muscle actin (α-SMA), but changes in vinculin or Snai2 protein amounts were not detectable in this tissue (Fig. 3C, D). Hereafter we used a single tamoxifen injection of 10 mg/ml (100 µl, total amount 1 mg per mouse) for all further experiments.

**Fig. 4:**
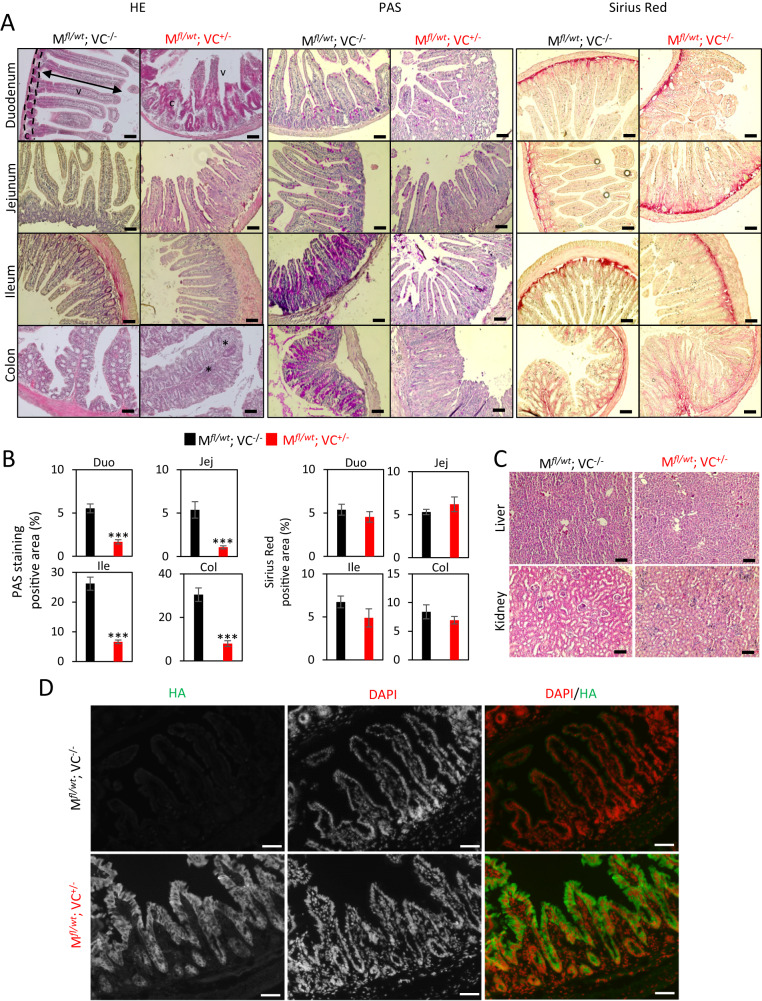
Cryptal hyperplasia and villus blunting upon MRTF-A activation in the gut. **A** Histological analysis of distinct intestinal segments by H&E staining, PAS staining for mucins, and Sirius Red staining for collagen-rich connective tissue. Compared are double-positive (M^*fl/wt*^ VC^+/−^) and control (M^*fl/wt*^ VC^−/−^) mice 7 days after one injection with 1 mg tamoxifen. Crypal zone (c) and villus zone (v) are indicated. Asterisks mark immune cell infiltrations. **B** Quantification of the areas positive for PAS- and Sirius Red staining. **C** H&E staining of liver and kidney of the indicated mice. **D** Immunofluorescence staining of cryosections of the small intestine, using anti-HA antibodies to detect constitutively active MRTF-A, and DAPI. Error bars, SEM (*n* = 3). ***p ≤ 0.001. Scale bars, 50 μm.

Prompted by the rapid and reversible weight loss, we suspected a transient malfunction of the intestinal epithelium by MRTF-A gain-of-function. Histological staining revealed an erosive architectural distortion with villus blunting, accompanied by marked cryptal hyperplasia, most prominently in the duodenum of the small intestine (Fig. 4A). An organized intestinal epithelium was hardly discernable by HE staining, and the brush border was absent. Similarly, the colon also showed erosive and architecturally distorted epithelia. While cryptal hyperplasia was less obvious, HE staining demonstrated epithelial expansion, edema and profound immune cell infiltration. The pathologies were partially reminiscent to those observed after intestinal injury, and in celiac or inflammatory bowel diseases. PAS staining was reduced in the intestines of double-positive mice, indicating depletion of goblet cells and impaired mucin production (Fig. 4A, B). No obvious fibrotic response was detectable by Sirius Red staining, consistent with the absence of intestinal shortening. In line with the absence of genetic recombination, kidney and liver were histologically unaffected, and were thus not implicated in the weight loss (Fig. 4C). Immunofluorescence of the small intestine confirmed the presence of the MRTF-A Δ3–5 protein in the crypts and along the epithelial cell layer of double-positive mice, mostly localizing to the cytoplasm (Fig. 4D). Together, the results suggest a transient severe damage of the intestinal epithelia, which is compensated by cryptal hyperplasia in the duodenum, and causes inflammation and leukocyte infiltration in the colon.

Sixty days after tamoxifen injection, only a weak recombination product could be amplified from different parts of the small and large intestine, suggesting that most cells expressing the MRTF-A gain-of-function allele were depleted (Supplementary Fig. S4A, B). However, a few recombined cells appear to have sustained and gave rise to isolated HA-positive crypt-villus structures, despite undectable levels of MRTF-A Δ3–5 in total protein extracts (Supplementary Fig. S4C, D). Inflammation was no longer observable at day 60, and length and weight of the intestine was unaltered (Supplementary Fig. S4E, F). Consistently, the intestines of injected mice showed a completely restored histology at day 60. Villus blunting, cryptal hyperplasia and immune cell infiltration had ceased, and PAS staining returned to normal (Supplementary Fig. S5). These results suggest that the activated MRTF-A Δ3–5 transiently impaired dynamic self-renewal of the intestinal epithelium, which is later repopulated mainly by non-recombined cells.

### Impaired growth and regeneration of transgenic mini-guts

**Fig. 5:**
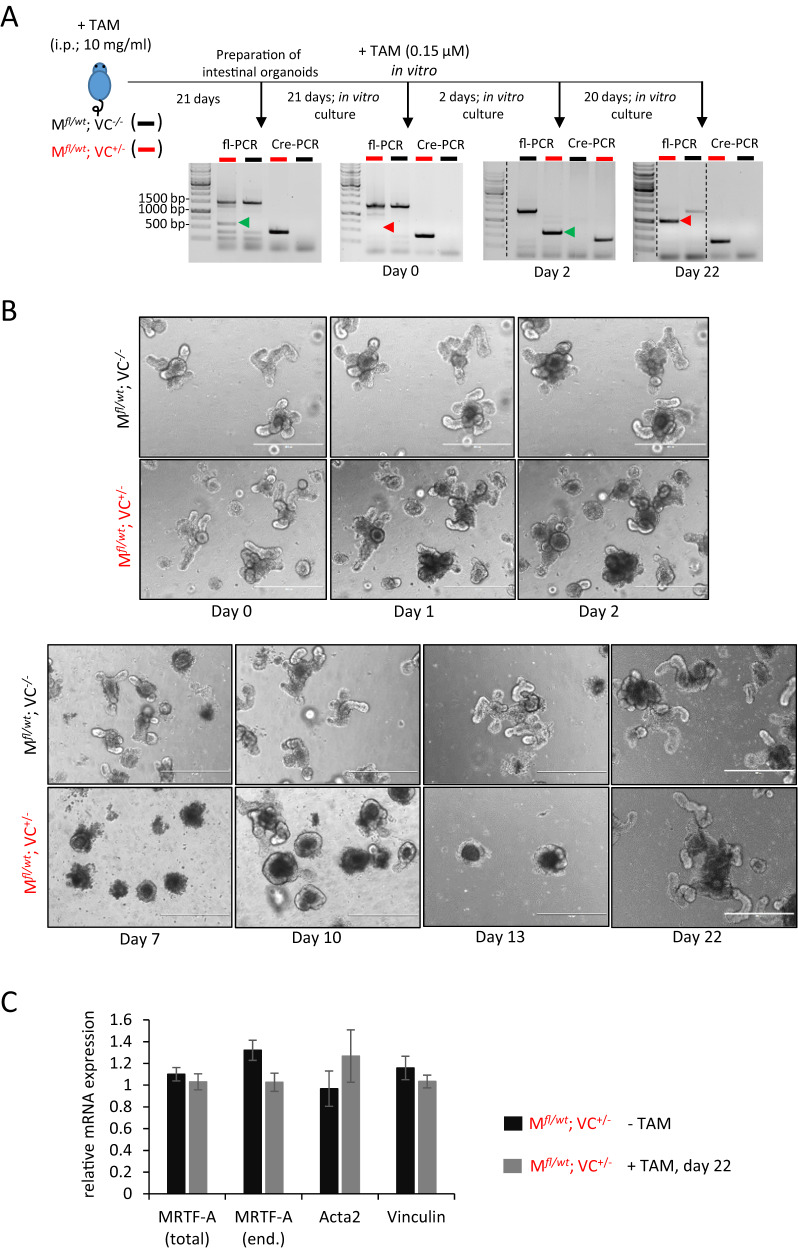
Intestinal epithelia with MRTF-A gain-of-function fail to form “mini-guts” and are repeatedly depleted from self-renewing 3D-structures. **A** Schematic of the experimental time scale to generate and treat intestinal organoids from the transgenic mice as indicated, with characterization by genotyping. Green arrowheads indicate a correctly recombined allele with deleted STOP cassette (ΔLSL), red arrowheads indicate its absence or an incorrectly rearranged transgene. **B** Phase contrast micrographs of viable 3D-organoids, with outward-bulging crypts, epithelial monolayers and a lumen darkened by shedded material. **C** Relative expression of the indicated mRNA in organoids from double-positive mice with and without tamoxifen treatment for 22 days, quantified by qRT-PCR and normalized to single-positive control organoids and housekeeping mRNA expression. Scale bars, 400 μm.

To get further insights into the epithelial recovery and turnover upon MRTF-A Δ3–5 expression, we analyzed the intestines of transgenic mice treated once with tamoxifen and generated intestinal organoids from these after 21 days. At this time point, genotyping showed only a very weak band of the Cre-induced recombination product in double-positive mice (green arrowhead in Fig. 5A), whereas the floxed allele dominated, indicating that the majority of cells expressing the activated MRTF-A Δ3–5 were depleted from the self-renewing epithelia (Fig. 5A). Intestinal organoids were prepared from the small intestine and cultivated for further 21 days in matrigel, allowing the formation of self-renewing “mini-guts”. Both double-positive and control organoids grew relatively normally, with outward-bulging crypts and some accumulation of darkly-visible material (presumably extruded enterocytes undergoing anoikis) in the inner lumen over time (Fig. 5B, day 0). At this time, there was no recombination product visible (red arrowhead in Fig. 5A). However, after recombination was then induced again by tamoxifen in vitro, increasing defects in growth and self-renewal were observed, especially after splitting of the organoids on day 2 after treatment (Fig. 5B). Genotyping confirmed a substantial depletion of the floxed allele and the concomitant appearance of the recombination product, lacking the STOP cassette (Fig. 5A).

Nevertheless, some double-positive organoids remained viable after tamoxifen treatment, but were round and lacked the outward-bulging crypts containing the stem cell compartment. The lumina appeared profoundly darkened, probably from accumulating dead cells. We kept cultivating these organoids for another 20 days and thereby coincidentally selected growth-competent organoids. The renewing capacity and the in vitro crypt formation eventually recovered and at day 22 of the experiment was indistinguishable from the control organoids. These re-growing organoids from double-positive mice lacked the expected recombination product, however. Instead, an aberrant recombination event had apparently occurred in the surviving cells, which resulted in a larger recombination product partially retaining the STOP cassette in front of the MRTF-A transgene (Fig. 5A, red arrowhead at day 22, and data not shown). Consistently, expression of MRTF-A and its target genes Acta2 (encoding smooth muscle actin) and vinculin in these organoids was unchanged compared to the control (Fig. 5C and data not shown). Together, this suggests that recovery of organoid growth in vitro required cells which escaped Cre-induced recombination, similar to the intestinal regeneration in vivo.

### MRTF-A Δ3–5 expression disrupts cryptal structure and function

**Fig. 6:**
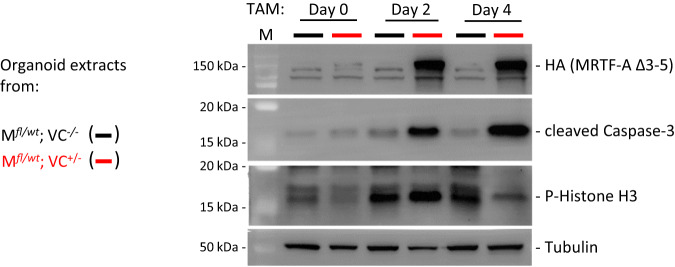
Altered caspase and phospho-histone levels in transgenic intestinal organoids upon tamoxifen induction ex vivo. Intestinal organoids were generated from the indicated transgenic mice, and cultured for 3 weeks before tamoxifen treatment. Shown is the immunoblotting of lysates from cultured organoids after 0, 2 and 4 days of tamoxifen treatment, using antibodies against HA, cleaved caspase-3, phospho-Histon H3 (P-H3), and tubulin as a control.

**Fig. 7:**
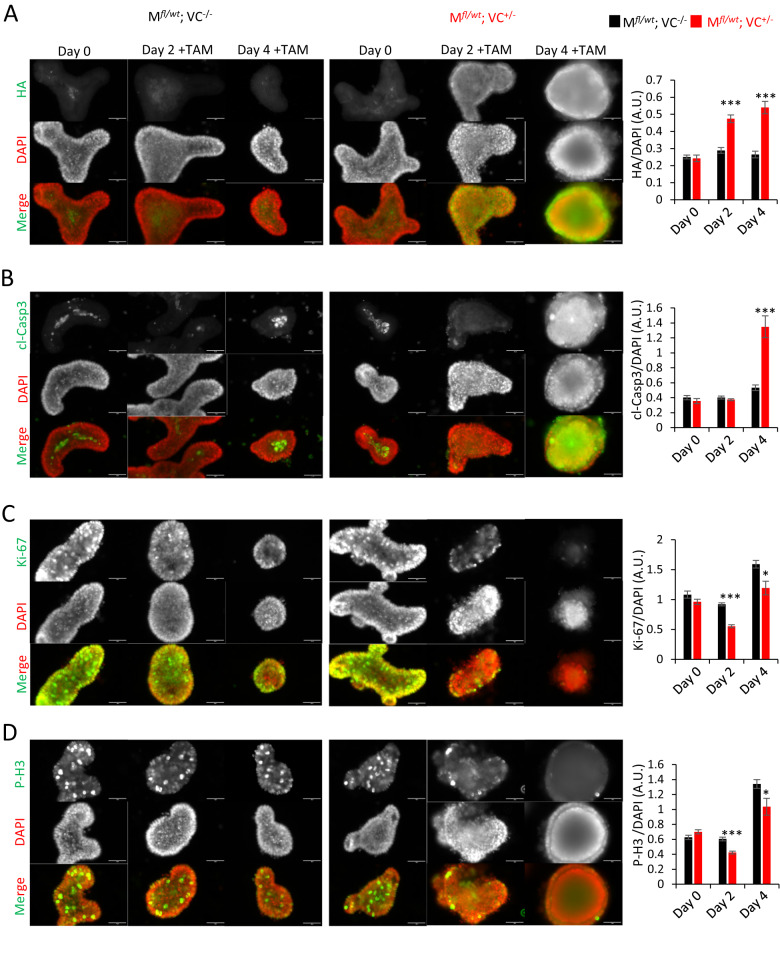
Apoptosis is increased, whilst proliferation markers are decreased in intestinal organoids expressing the activated MRTF-A Δ3–5. **A** Organoids generated from the indicated transgenic mice were cultured for 3 weeks and then treated with 0.15 µM tamoxifen before immunofluorescence staining using antibodies against HA (**A**), cleaved caspase-3 (cl-Casp3; **B**), Ki-67 (**C**) and phospho-Histon H3 (P-H3; **D**), as indicated. Nuclei were counterstained with DAPI and are false-colored in red. Relative signal intensity normalized to DAPI is shown on the right of each panel. Error bars, SEM (*n* = 3). *p ≤ 0.05, **p ≤ 0.01, ***p ≤ 0.001. Scale bars, 50 μm.

To better understand the nature of the defect caused by MRTF-A Δ3–5, we freshly isolated organoids from single-positive and double-positive mice and activated MRTF-A Δ3–5 after three weeks of culture. Following two and four days of tamoxifen treatment, MRTF-A Δ3–5 protein was readily detectable in protein extracts of double-positive organoids (Fig. 6). Concomitantly, we observed an increase in cleaved caspase-3 and a decrease of the proliferation marker phospho-Histone H3 (Fig. 6). Analysis by immunofluorescence microscopy confirmed that HA-tagged MRTF-A Δ3–5 was expressed throughout the double-positive organoids within two to four days (Fig. 7A). Apoptotic cells positive for cleaved caspase-3 were visible within the lumen of control organoids, whilst the intensity of cleaved caspase-3 was increased at day 4 in double-positive organoids (Fig. 7B). The proliferation marker Ki-67 and the mitotic marker phospho-Histone H3 was visible in distinct cells of the epithelia, whose number and overall fluorescence intensity decreased in double-positive organoids (Fig. 7C, D). This suggested aberrant apoptosis simultaneously to a decreased proliferation rate in epithelia with MRTF-A gain-of-function.

**Fig. 8:**
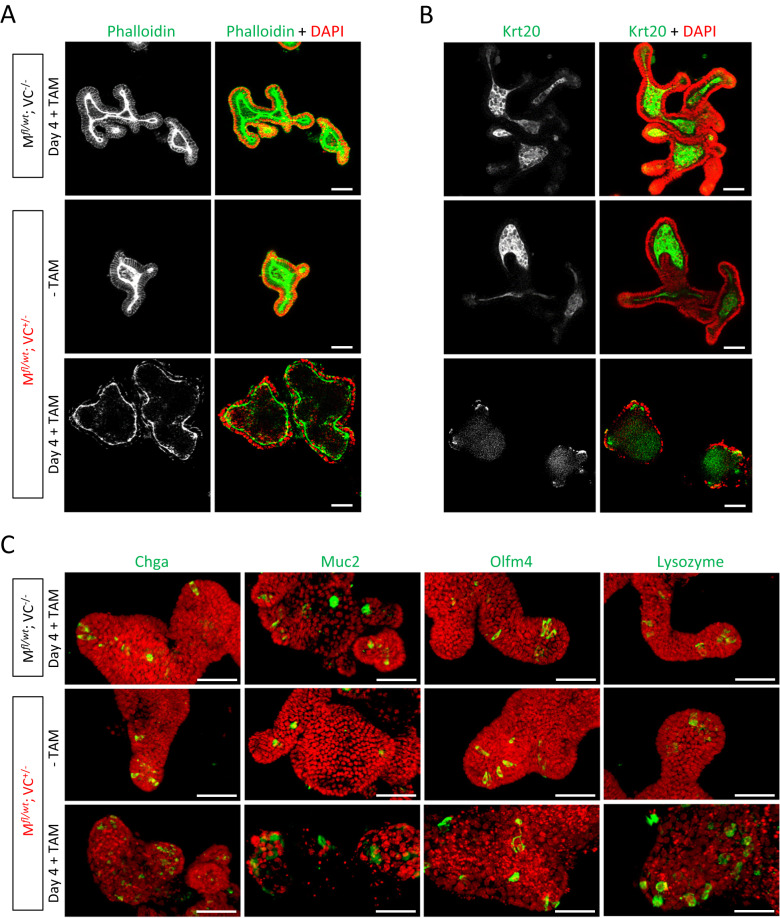
Differentiation, polarization and localization of cell types in transgenic intestinal organoids. Immunofluorescent images of intestinal organoid cultures from the indicated mice, treated for 4 days with 0.15 µM tamoxifen (+TAM) or without (−TAM). **A** Single optical sections of whole-mount organoids stained for F-actin by phalloidin (green) and DAPI (red) for nuclei, using ApoTome2. Representative images show F-actin mislococalisation and reduction, nuclear fragmentation and altered organoid architecture upon MRTF-A activation. **B** Immune staining of the enterocyte marker protein cytokeratin 20 (Krt20, green). **C** 3D maximum projection images of organoids using ApoTome2 z-stacks. Immune staining (in green) was performed for markers of enterochromaffine cells (Chga, Chromogranin A), goblet cells (Muc2, Mucin2), stem cells (Olfm4, Olfactomedin 4) and paneth cells (Lysozyme). Nuclei were counterstained with DAPI and are false-colored in red. Scale bars, 50 μm.

Optical sections of phalloidin-stained control organoids showed a pronounced apical F-actin belt lining the lumen and weaker basolateral actin filaments surrounding the columnar-shaped cells (Fig. 8A). After tamoxifen-induced MRTF-A gain-of-function, phalloidin intensity, cell polarization and outward-bulging crypts were decreased. Concomitantly, immunostaining of cytokeratin 20, a marker for differentiated enterocytes located mostly in villi, was also reduced (Fig. 8B). To visualize particular cell types present in intestinal organoids, 3D-reconstructed images of whole-mount organoids stained for Chga (enterochromaffine cells), Muc2 (goblet cells), Olfm4 (stem cells) and lysozyme (paneth cells) were analyzed. Whilst positively stained cells were easily detectable in single-positive und untreated control organoids, tamoxifen-induced double-positive organoids showed altered localization and integrity of these cell types (Fig. 8C). Of note, Olfm4-positive stem cells were reduced and lysozyme-positive paneth cells appeared to localize randomly. Together, this indicated that polarization, differentiation and crypt architecture is affected by MRTF-A Δ3–5.

**Fig. 9:**
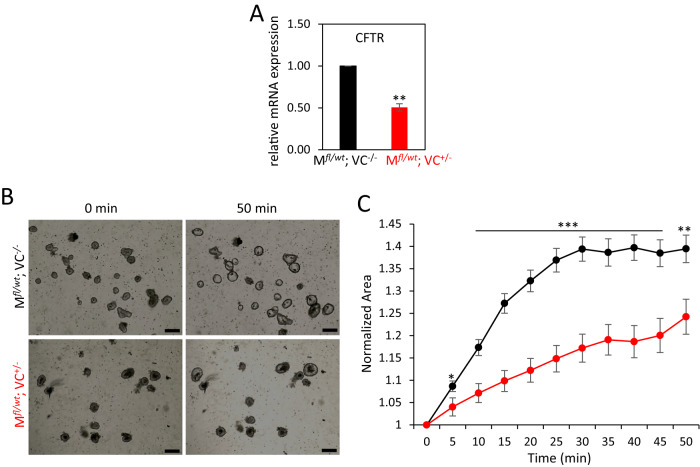
Impaired CFTR expression and organoid swelling upon MRTF-A activation. **A** Relative expression of CFTR mRNA in organoids from control and double-positive mice three days after treatment with tamoxifen (0.15 µM), quantified by qRT-PCR and normalized to housekeeping mRNA expression. **B** Representative phase contrast micrographs of organoids at the indicated time points during forskolin-induced (10 µM) swelling experiment. **C** Normalized organoid area over the time of forskolin treatment. Error bars, SEM (*n* = 10). **p ≤ 0.01, ***p ≤ 0.001. Scale bars, 50 μm.

To test whether the cryptal compartment is particularly affected by MRTF-A gain-of-function, we analyzed the cAMP-dependent chloride channel CFTR, which is predominantly expressed in the crypt [41]. Firstly, we investigated CFTR mRNA expression and found that it was reduced by around 50% in tamoxifen-treated organoids from double-positive mice (Fig. 9A). Moreover, CFTR mediated swelling of the organoids by forskolin treatment was functionally affected. Time-course analysis revealed that the swelling and the volume increase was abated in organoids expressing MRTF-A Δ3–5 (Fig. 9B, C). This suggests that the cryptal compartment, which harbors the stem cell population and is responsible for organoid self-renewal, is particularly harmed by MRTF-A gain-of-function. In addition, MRTF-A Δ3–5 may also functionally affect intestinal physiology via CFTR reduction.

### Altered expression of targets and cell type markers

**Fig. 10:**
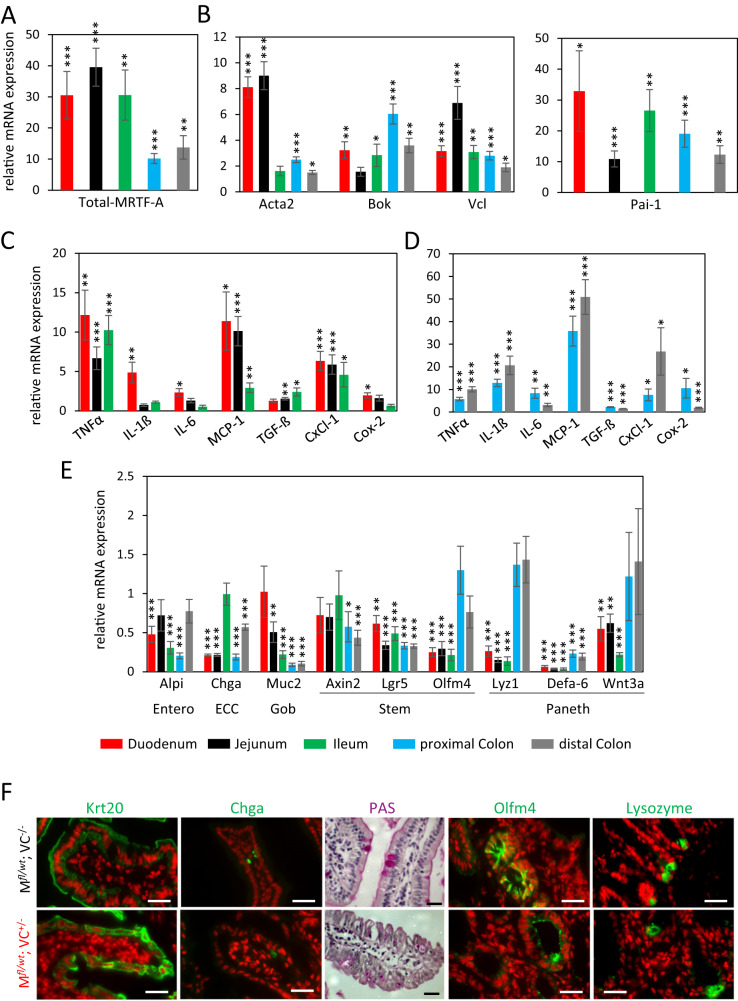
Relative changes in gene expression in the mouse intestine upon MRTF-A gain-of-function at day 7 after tamoxifen treatment. RNA was prepared from the five indicated intestinal segments of three double-positive and three control mice, all injected once with 1 mg tamoxifen. qRT-PCR was performed with intron-spanning primer pairs for the genes indicated, and normalized to housekeeping control genes. Bar colors represent the duodenum, jejunum, ileum, proximal colon and distal colon, as indicated. **A** Relative mRNA of MRTF-A transcripts (including the transgenic) in comparison to the single-positive control mice. **B** MRTF-A target genes *Acta2*, *Bok*, *Vcl* and *Pai-1*. **C** Inflammatory marker genes in the small intestine. **D** Inflammatory marker genes in the large intestine. **E** Marker genes for intestinal cell types as indicated underneath. Entero, enterocytes; ECC, enterochromaffine cells; Gob, goblet cells; Stem, stem cells; Paneth, paneth cells. **F** Cell type-specific staining of villi (Krt20, Chga) and crypts (Olfm4, Lysozyme) of the small intestine of double-positive and control mice using immunofluorescence against the indicated proteins (green) or PAS (violet). Nuclei were counterstained with DAPI and are false-colored in red, except for PAS staining. Error bars, SEM (*n* = 3). *p ≤ 0.05, **p ≤ 0.01, ***p ≤ 0.001. Scale bars, 30 μm.

As a transcription factor, MRTF-A controls mRNA expression of various target genes, and can thereby affect the differentiation of epithelial lineages. Thus, we analyzed relative mRNA amounts in the duodenum, jejunum, ileum, proximal and distal colon from mice sacrificed 7 days after tamoxifen injection. Firstly, the upregulation of total MRTF-A mRNA in comparison to control mice was confirmed by qRT-PCR (Fig. 10A). Secondly, the previously characterized target genes Bok, Vcl and Acta2 were also upregulated, the latter one most prominently in duodenum and jejunum (Fig. 10B). Moreover, expression of Pai-1 (serpine1), which we previously characterized as a G-actin-regulated putative MRTF-A target gene in fibroblasts, was induced between 10 and 30 fold in different intestinal segments (Fig. 10B) [31, 42].

Since some myosin family members are known MRTF-SRF targets (Myo1C, Myh9, Myh14) and are involved in microvilli-dependent villus atrophy, we also tested expression of myosins. As yet, the mRNA of Myo5B, Myo1A and Myh14 appeared slightly reduced in some regions of the gut, whilst Myo1C and Myh9 were moderately elevated at day 7 (Supplementary Fig. S6). However, the generally weak effects did not proove a direct or causal role of deregulated myosins in the observed pathologies.

Given that we showed increased leukocyte infiltration in HE-stained large intestines, we quantified the expression of pro-inflammatory cytokines. TNFalpha, MCP-1 and Cxcl-1 mRNA were increased in the small intestine around 5–10 fold after 7 days (Fig. 10C). In the proximal and distal colon, however, all cytokine mRNAs were upregulated, many of them to a much higher extent (Fig. 10D). This suggests an early inflammatory process in the double-positive mice, probably caused by destruction of the epithelial architecture following the impaired self-renewal process upon aberrant MRTF-A gain-of-function.

Next, we analyzed expression of marker genes specific for enterocytes, enterochromaffine cells, goblet cells, stem cells and paneth cells. Overall, most marker genes were significantly reduced in the five intestinal segments analyzed, albeit to a different extent (Fig. 10E). The goblet cell marker Muc2 was particularly attenuated in proximal and distal sections of the colon. Paneth cell markers Lyz1, Defa-6 and Wnt3a were most prominently reduced in all parts of the small intestine, whereas they showed less or no changes in the colon. Axin2, Lgr5 and Olfm4, which are considered as stem cell markers, were downregulated in most parts of the intestine. A similar albeit smaller reduction in most cell type-specific marker expression was also observed in the intestinal organoids treated with tamoxifen in vitro, simultaneous to the increase in MRTF-A target genes (Supplementary Fig. S7). Specifically, Lgr5 was significantly reduced by 40–70% in all intestinal segments of the double positve mice. Considering that cryptal Lgr5-positive stem cells are required to rapidly replenish the highly plastic intestinal epithelium, including all other more specialized cell types, this reduction may explain some of the defects observed.

Finally, we immunostained the small intestine of the transgenic mice to visualize the number and localization of selected cell types. In areas where the epithelial architecture was still sufficiently maintained for assessment, MRTF gain-of-function mice showed partially detaching cells with little specific Krt20 localization, whilst in control mice Krt20 localized apically in polarized villus enterocytes (Fig. 10F). The numbers of Chga-positive enterochromaffine cells and PAS-positive goblet cells were strongly reduced. In the crypts, Olfm4-positive stem cells and lysozyme-positive paneth cells were not only strongly reduced in numbers, but also mislocated, and the typical alternating pattern of the two cell types was absent in double-positive intestines (Fig. 10F). These results indicate that physiological renewal from the cryptal stem cell compartment and differentiation into villus epithelial cells is impaired by MRTF-A gain-of-function in vivo at day 7 after induction. At day 60, however, expression of cell type markers and cytokines had reversed to normal, in line with the resolved phenotype, and only a slight increase of MRTF-A and Acta2 expression mostly in the small intestine was still detectable (Supplementary Fig. S8). Together, the data suggest that genetic MRTF-A activation transiently impaired stem cell-driven renewal of the intestinal epithelium and caused apoptotic injury of villus cells, followed by inflammation. Resolving the phenotype occurred due to cryptal hyperplasia and repopulation with cells which had escaped MRTF-A Δ3–5 expression after the single tamoxifen dose.

## Discussion

The intestinal epithelium is a highly dynamic tissue which is constantly and rapidly undergoing self-renewal from cryptal stem cells. In a genetic mouse model, we show here that inducible MRTF-A gain-of-function impairs the delicate balance between proliferation, differentiation and apoptosis. This affected most of its cell types, including the stem cells. The intestinal epithelium was thus severely damaged by activated MRTF-A expression and acute villus atrophy, inflammation and cryptal hyperplasia was observed. An “adaptive differentiation model” of atrophy-induced villus epithelial cells (aVEC) expressing fetal markers was recently proposed to repair acute injuries [29]. Whether the rapid restoration of the barrier in our mouse model occurs via these aVEC needs to be clarified, but we note no upregulation of stem cell markers at day 7. Nevertheless, we observed cryptal hyperplasia, a well-described compensatory mechanism upon injury, which may be driven by inflammation and submucosal growth factor release.

MRTF activity was recently implicated in intestinal fibrosis associated with IBD and myofibroblast-mediated gut shortening [43–45]. Using the Villin-CreERT2 model, we did not see obvious fibrotic responses neither in acutely affected animals nor at later time points after MRTF-A activation. This supports the hypothesis that a fibrotic response is predominantly dependent on mesenchymal fibroblasts within the intestine, and that there is little transdifferentiation between cell lineages. Secondly, the rapid depletion of most of the recombined intestinal epithelial cells likely prevented a persistent induction of EMT, epithelial-myofibroblast transition, or a subsequent establishment of a profibrotic epithelial phenotype, as it was demonstrated for other epithelia via involvement of MRTF-A [16–21]. In line, we did not observe Snai2 upregulation in the affected intestine.

After one tamoxifen injection, the fast recovery of our mice after 7 days was apparently driven by the intestinal cells which had escaped Cre-mediated deletion of the STOP cassette and replenished the stem cell pool. Repeated tamoxifen injections which exacerbate the activity of the Cre recombinase caused a lethal intestinal malfunction (not shown), suggesting a mosaic expression of MRTF-A Δ3–5 after only one tamoxifen injection. It needs to be clarified whether the repair and recovery occurred by dynamic dedifferentiation of multiple cell types or by surviving reserve stem cells [22]. However, some recombined cells and elevations of MRTF-A and Acta2 expression were still evident in the small intestine after 60 days, when phenotype and histology have largely reverted to normal. Whether this has consequences on susceptibility for small bowel enteropathies like IBD remains to be investigated.

Mechanistically, increased apoptosis and decreased proliferation was implicated in MRTF-A induced epithelial damage, in line with previous observations in cultured cells [31, 32]. Amongst others, the intestinal stem cells and their multifunctional guardians, the paneth cells, were partially depleted upon MRTF-A activation, as judged by reduced expression of their markers, the lack of cryptal outgrowth in vitro and the organoid swelling experiments. The (transient) depletion of the intestinal stem cells, which are known to be particularly susceptible to damage, decreases all other cell types and shifts the balance from renewal to death [22]. Moreover, the anti-proliferative effect of MRTF-A may also impair the division of the transit amplifying cells.

Following an upward movement, intestinal cells physiologically die of anoikis after extrusion at the tips of the villi. A very elegant paper recently showed that this movement, in addition to the pushing forces by mitotic pressure from the crypt, requires active Arp2/3 dependent tensile migratory forces in villi [46]. Additionally, the strength of cell contacts and actomyosin activity also enables the collective upward migration in the crypt [47]. MRTF-A is a master regulator of mechanotransduction and directly controls expression of many cytoskeletal and cell adhesion components such as myosins, actins, integrins and vinculin [4, 42]. Thus, we speculate that the MRTF-A activation in the intestinal epithelial cells may also affect the upward movement and hence steady-state turnover. Whether this acts in addition to, or is even causative for, the induction of apoptosis in our model remains to be determined. Intriguingly, the epithelial cells of the small intestine in MRTF-A gain-of-function mice showed altered morphology, loss of brush border, and lack of polarized columnal epithelial architecture, which suggests changes in their actin cytoskeleton.

For our gain-of-function mouse model, we generated a novel constitutively active MRTF-A construct by deleting sequences corresponding to the murine exons 3–5, which encode amino acids 81–146 in the N-terminal RPEL domain (according to murine MRTF-A full length, transcript variant 2 with 14 exons and 1029 amino acids, NCBI NM_001082536.2 and NP_001076005.2) [3, 8, 48]. This domain contains three 22-amino acids RPEL motifs [7]. Truncation of the entire N-terminus is known to render the protein constitutively active (MRTF-A ΔN) [8]. The 3 RPEL motifs are separated by short spacers and both, the RPEL motifs as well as the intervening spacers, have been shown to bind specifically to G-actin. The actin-binding capabilities of RPEL1 (exon 3), RPEL2 (exon 5) and the intervening spacer1 as a trimeric complex control the extracellular signal-induced subcellular localization and transcriptional activity of MRTF-A, as these domains exhibit a high binding-affinity towards G-actin [9, 49]. In contrast, the binding of RPEL3 (exon 6) to actin is less stable. This suggested that actin-binding to RPEL1, RPEL2, and spacer1 - rather than to RPEL3 and spacer2 - is required for the repression of MRTF-A’s transcriptional activity. In vitro testing confirmed that our MRTF-A Δ3–5 is indeed constitutively active, with intermediate activity between MRTF-A full length and MRTF-A ΔN. Using this construct for a conditional transgenic mouse, we generated a unique MRTF gain-of-function model which helps to better understand the in vivo consequences of elevated MRTF activity. Limitations of our approach are, however, the maintained endogenous MRTF-A locus and the elevated expression level of the transgene driven by an unrelated promoter, as demonstrated in comparison to total MRTF-A in control mice.

Serum response factor (SRF), the transcription factor primarily targeted by MRTFs, has previously been analyzed in gain-of-function mice by the Nordheim lab. A SRF-VP16 construct caused spontaneous hepatocellular carcinoma after 25–40 weeks [50]. This carcinogenic effect is thought to be primarily caused by MRTF-independent, TCF-dependent pro-proliferative target genes [5]. In contrast, we observed here upon ubiquitous MRTF gain-of-function a massive architectural distortion and ballooning of the liver, reminiscent of acute liver intoxication, which was not reported for SRF-VP16 transgenic mice. While we do not know the underlying mechanism, we note that the speed of this lethal effect is, to our knowledge, unprecedented for an inducible transgene. The described phenotype is unlikely explainable by hepatic stellate cell-mediated liver fibrosis alone [51]. Unfortunately, speed and lethality prevented thorough analysis concerning carcinogenic effects of ubiquitous MRTF-A activation in mice, but we did not observe obvious nodules in the liver, neither in the intestine of Villin-CreERT2 breedings. In embryonic stem cells, SRF-VP16 causes a massive increase of stress fibers and lamellipodia [52]. This is consistent with previous observations of MRTF-A affecting the cytoskeletal architecture through a subset of SRF target genes [4, 31, 42]. We propose that this may contribute to some of the disturbances in our MRTF gain-of-function mice, including the lethality upon embryonic and ubiquitous activation of MRTF-A. For example, embryonic lethality may occur as early as in gastrulation, in line with the previously shown phenotypes of SRF knockouts [15, 52].

MRTF-A is readily expressed in most tissues, including the small and large intestine of the mouse [30]. The absence of an obvious intestinal phenotype in the knockout does not preclude a pathological effect upon aberrant activation, as we showed here. Several studies have implicated high Rho-actin-MRTF-SRF signaling with fibrotic response in various tissues, including the gut during IBD such as Crohn’s and colitis [43–45]. The rapid and pronounced intestinal phenotype we describe for the genetic MRTF-A gain-of-function does not permit chronic autoinflammatory responses, but harbors similar characteristics like villi shortening, reduced brush border zone and loss of epithelial markers. Interestingly, we previously identified Pai-1 (Serpine1) as a G-actin-regulated MRTF/SRF target gene [31, 42]. Pai-1 was also upregulated in the MRTF-A gain-of-function intestinal epithelium. Recently, elevated PAI-1 has been shown to augment mucosal damage in IBD by linking the epithelium to a pro-inflammatory immune response [53]. This raises the possibility that both mesenchymal and epithelial MRTF-A plays a role in enteropathies and explains how initial tissue stiffening can become auto-propagative via elevated levels of PAI-1. Thus, an intestine-specific MRTF-A gain-of-function mouse model might be useful to better understand intestinal injury, repair and regeneration.

### Supplementary information

Supplemental Figures S1-S8 and Table S1

Fullsize uncropped western blots

## Data Availability

Upon reasonable request, details and materials will be made available.

## References

[1] Olson EN, Nordheim A (2010). Linking actin dynamics and gene transcription to drive cellular motile functions. Nat Rev Mol Cell Biol.

[2] Posern G, Treisman R (2006). Actin’ together: serum response factor, its cofactors and the link to signal transduction. Trends Cell Biol.

[3] Reed F, Larsuel ST, Mayday MY, Scanlon V, Krause DS (2021). MRTFA: a critical protein in normal and malignant hematopoiesis and beyond. J Biol Chem.

[4] Esnault C, Stewart A, Gualdrini F, East P, Horswell S, Matthews N (2014). Rho-actin signaling to the MRTF coactivators dominates the immediate transcriptional response to serum in fibroblasts. Genes Dev.

[5] Gualdrini F, Esnault C, Horswell S, Stewart A, Matthews N, Treisman R (2016). SRF co-factors control the balance between cell proliferation and contractility. Mol Cell.

[6] Baarlink C, Wang H, Grosse R (2013). Nuclear actin network assembly by formins regulates the SRF coactivator MAL. Science.

[7] Guettler S, Vartiainen MK, Miralles F, Larijani B, Treisman R (2008). RPEL motifs link the serum response factor cofactor MAL but not myocardin to Rho signaling via actin binding. Mol Cell Biol.

[8] Miralles F, Posern G, Zaromytidou AI, Treisman R (2003). Actin dynamics control SRF activity by regulation of its coactivator MAL. Cell.

[9] Mouilleron S, Langer CA, Guettler S, McDonald NQ, Treisman R (2011). Structure of a pentavalent G-actin*MRTF-A complex reveals how G-actin controls nucleocytoplasmic shuttling of a transcriptional coactivator. Sci Signal.

[10] Vartiainen MK, Guettler S, Larijani B, Treisman R (2007). Nuclear actin regulates dynamic subcellular localization and activity of the SRF cofactor MAL. Science.

[11] Li J, Zhu X, Chen M, Cheng L, Zhou D, Lu MM (2005). Myocardin-related transcription factor B is required in cardiac neural crest for smooth muscle differentiation and cardiovascular development. Proc Natl Acad Sci USA.

[12] Li S, Chang S, Qi X, Richardson JA, Olson EN (2006). Requirement of a myocardin-related transcription factor for development of mammary myoepithelial cells. Mol Cell Biol.

[13] Oh J, Richardson JA, Olson EN (2005). Requirement of myocardin-related transcription factor-B for remodeling of branchial arch arteries and smooth muscle differentiation. Proc Natl Acad Sci USA.

[14] Sun Y, Boyd K, Xu W, Ma J, Jackson CW, Fu A (2006). Acute myeloid leukemia-associated Mkl1 (Mrtf-a) is a key regulator of mammary gland function. Mol Cell Biol.

[15] Arsenian S, Weinhold B, Oelgeschlager M, Ruther U, Nordheim A (1998). Serum response factor is essential for mesoderm formation during mouse embryogenesis. EMBO J.

[16] Busche S, Descot A, Julien S, Genth H, Posern G (2008). Epithelial cell-cell contacts regulate SRF-mediated transcription via Rac-actin-MAL signalling. J Cell Sci.

[17] Fan L, Sebe A, Peterfi Z, Masszi A, Thirone AC, Rotstein OD (2007). Cell contact-dependent regulation of epithelial-myofibroblast transition via the rho-rho kinase-phospho-myosin pathway. Mol Biol Cell.

[18] Miranda MZ, Lichner Z, Szaszi K, Kapus A (2021). MRTF: basic biology and role in kidney disease. Int J Mol Sci.

[19] Morita T, Mayanagi T, Sobue K (2007). Dual roles of myocardin-related transcription factors in epithelial mesenchymal transition via slug induction and actin remodeling. J Cell Biol.

[20] Seifert A, Posern G (2017). Tightly controlled MRTF-A activity regulates epithelial differentiation during formation of mammary acini. Breast Cancer Res.

[21] Bialik JF, Ding M, Speight P, Dan Q, Miranda MZ, Di Ciano-Oliveira C (2019). Profibrotic epithelial phenotype: a central role for MRTF and TAZ. Sci Rep.

[22] Beumer J, Clevers H (2021). Cell fate specification and differentiation in the adult mammalian intestine. Nat Rev Mol Cell Biol.

[23] Barker N, van Es JH, Kuipers J, Kujala P, van den Born M, Cozijnsen M (2007). Identification of stem cells in small intestine and colon by marker gene Lgr5. Nature.

[24] Sato T, van Es JH, Snippert HJ, Stange DE, Vries RG, van den Born M (2011). Paneth cells constitute the niche for Lgr5 stem cells in intestinal crypts. Nature.

[25] Snippert HJ, van der Flier LG, Sato T, van Es JH, van den Born M, Kroon-Veenboer C (2010). Intestinal crypt homeostasis results from neutral competition between symmetrically dividing Lgr5 stem cells. Cell.

[26] Jansson-Knodell CL, Hujoel IA, Rubio-Tapia A, Murray JA (2018). Not all that flattens villi is celiac disease: a review of enteropathies. Mayo Clin Proc.

[27] Ayyaz A, Kumar S, Sangiorgi B, Ghoshal B, Gosio J, Ouladan S (2019). Single-cell transcriptomes of the regenerating intestine reveal a revival stem cell. Nature.

[28] Murata K, Jadhav U, Madha S, van Es J, Dean J, Cavazza A (2020). Ascl2-dependent cell dedifferentiation drives regeneration of ablated intestinal stem cells. Cell Stem Cell.

[29] Ohara TE, Colonna M, Stappenbeck TS (2022). Adaptive differentiation promotes intestinal villus recovery. Dev Cell.

[30] Wang DZ, Li S, Hockemeyer D, Sutherland L, Wang Z, Schratt G (2002). Potentiation of serum response factor activity by a family of myocardin-related transcription factors. Proc Natl Acad Sci USA.

[31] Descot A, Hoffmann R, Shaposhnikov D, Reschke M, Ullrich A, Posern G (2009). Negative regulation of the EGFR-MAPK cascade by actin-MAL-mediated Mig6/Errfi-1 induction. Mol Cell.

[32] Shaposhnikov D, Descot A, Schilling J, Posern G (2012). Myocardin-related transcription factor A regulates expression of Bok and Noxa and is involved in apoptotic signalling. Cell Cycle.

[33] Platt RJ, Chen S, Zhou Y, Yim MJ, Swiech L, Kempton HR (2014). CRISPR-Cas9 knockin mice for genome editing and cancer modeling. Cell.

[34] Betz UA, Vosshenrich CA, Rajewsky K, Muller W (1996). Bypass of lethality with mosaic mice generated by Cre-loxP-mediated recombination. Curr Biol.

[35] Hameyer D, Loonstra A, Eshkind L, Schmitt S, Antunes C, Groen A (2007). Toxicity of ligand-dependent Cre recombinases and generation of a conditional Cre deleter mouse allowing mosaic recombination in peripheral tissues. Physiol Genomics.

[36] el Marjou F, Janssen KP, Chang BH, Li M, Hindie V, Chan L (2004). Tissue-specific and inducible Cre-mediated recombination in the gut epithelium. Genesis.

[37] O’Rourke KP, Dow LE, Lowe SW (2016). Immunofluorescent staining of mouse intestinal stem cells. Bio Protoc.

[38] Werner S, Lutzkendorf J, Muller T, Muller LP, Posern G (2019). MRTF-A controls myofibroblastic differentiation of human multipotent stromal cells and their tumour-supporting function in xenograft models. Sci Rep.

[39] Pfaffl MW (2001). A new mathematical model for relative quantification in real-time RT-PCR. Nucleic Acids Res.

[40] Panayiotou R, Miralles F, Pawlowski R, Diring J, Flynn HR, Skehel M (2016). Phosphorylation acts positively and negatively to regulate MRTF-A subcellular localisation and activity. Elife.

[41] Singh AK, Riederer B, Krabbenhoft A, Rausch B, Bonhagen J, Lehmann U (2009). Differential roles of NHERF1, NHERF2, and PDZK1 in regulating CFTR-mediated intestinal anion secretion in mice. J Clin Invest.

[42] Leitner L, Shaposhnikov D, Mengel A, Descot A, Julien S, Hoffmann R (2011). MAL/MRTF-A controls migration of non-invasive cells by upregulation of cytoskeleton-associated proteins. J Cell Sci.

[43] Holvoet T, Devriese S, Castermans K, Boland S, Leysen D, Vandewynckel YP (2017). Treatment of intestinal fibrosis in experimental inflammatory bowel disease by the pleiotropic actions of a local rho kinase inhibitor. Gastroenterology.

[44] Johnson LA, Rodansky ES, Haak AJ, Larsen SD, Neubig RR, Higgins PD (2014). Novel Rho/MRTF/SRF inhibitors block matrix-stiffness and TGF-beta-induced fibrogenesis in human colonic myofibroblasts. Inflamm Bowel Dis.

[45] Johnson LA, Rodansky ES, Sauder KL, Horowitz JC, Mih JD, Tschumperlin DJ (2013). Matrix stiffness corresponding to strictured bowel induces a fibrogenic response in human colonic fibroblasts. Inflamm Bowel Dis.

[46] Krndija D, El Marjou F, Guirao B, Richon S, Leroy O, Bellaiche Y (2019). Active cell migration is critical for steady-state epithelial turnover in the gut. Science.

[47] Perez-Gonzalez C, Ceada G, Greco F, Matejcic M, Gomez-Gonzalez M, Castro N (2021). Mechanical compartmentalization of the intestinal organoid enables crypt folding and collective cell migration. Nat Cell Biol.

[48] Scharenberg MA, Pippenger BE, Sack R, Zingg D, Ferralli J, Schenk S (2014). TGF-beta-induced differentiation into myofibroblasts involves specific regulation of two MKL1 isoforms. J Cell Sci.

[49] Mouilleron S, Guettler S, Langer CA, Treisman R, McDonald NQ (2008). Molecular basis for G-actin binding to RPEL motifs from the serum response factor coactivator MAL. EMBO J.

[50] Ohrnberger S, Thavamani A, Braeuning A, Lipka DB, Kirilov M, Geffers R (2015). Dysregulated serum response factor triggers formation of hepatocellular carcinoma. Hepatology.

[51] Shi Z, Ren M, Rockey DC (2020). Myocardin and myocardin-related transcription factor-A synergistically mediate actin cytoskeletal-dependent inhibition of liver fibrogenesis. Am J Physiol Gastrointest Liver Physiol.

[52] Schratt G, Philippar U, Berger J, Schwarz H, Heidenreich O, Nordheim A (2002). Serum response factor is crucial for actin cytoskeletal organization and focal adhesion assembly in embryonic stem cells. J Cell Biol.

[53] Kaiko GE, Chen F, Lai CW, Chiang IL, Perrigoue J, Stojmirovic A (2019). PAI-1 augments mucosal damage in colitis. Sci Transl Med.

