# Supplementary material for: MRTF-A gain-of-function in mice impairs homeostatic renewal of the intestinal epithelium
Source: Cell Death Dis. 2023 Sep 28;14(9):639. doi: 10.1038/s41419-023-06158-4 (PMC10539384; doi:10.1038/s41419-023-06158-4)
Supplement: Supplementary file 1 — Supplemental Figures S1-S8 and Table S1 [file 41419_2023_6158_MOESM1_ESM.pdf]

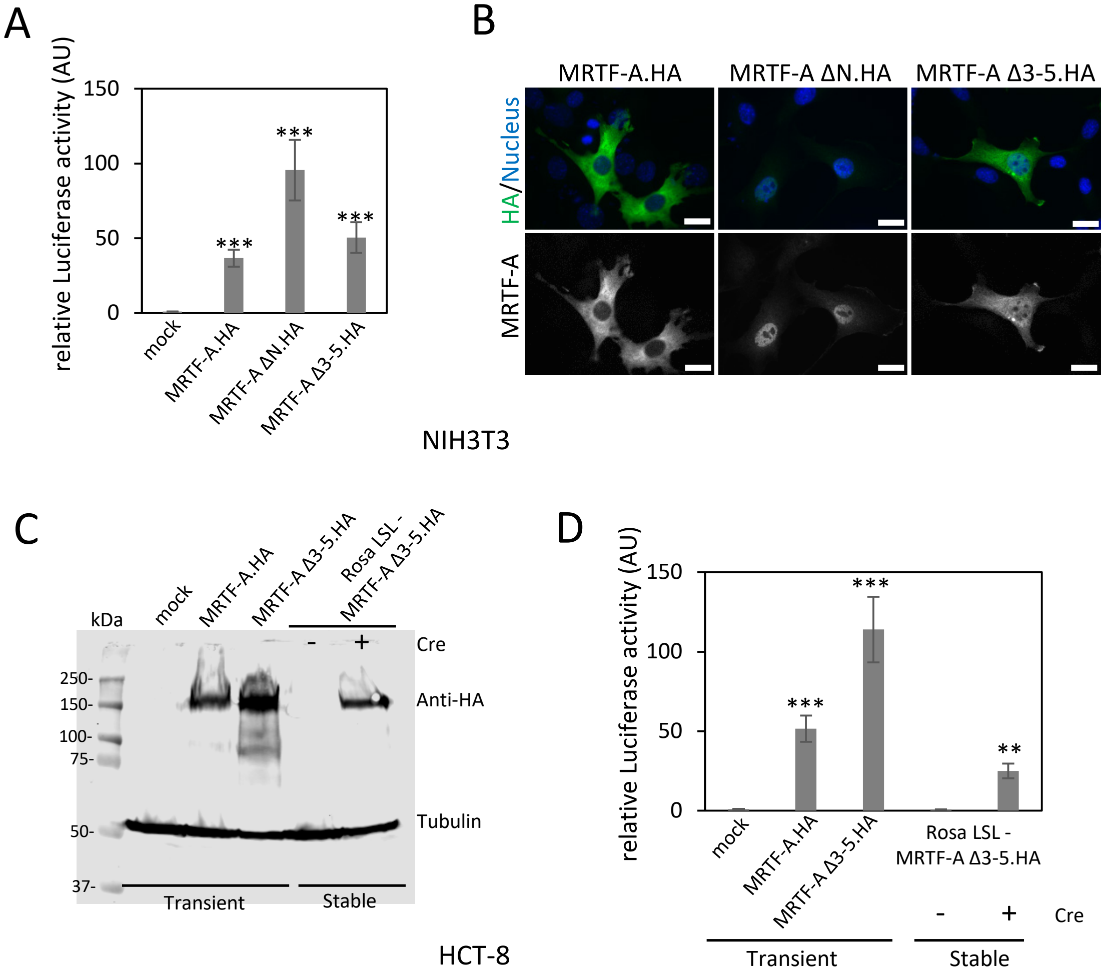

Supplementary Figure S1: Functional characterization of MRTF-A Δ3-5. (A) NIH3T3 cells were transiently transfected with cDNA expression plasmids resembling the desired deletion of exon 3-5, compared to ΔN and full length. Shown is the relative activation of the MRTF/SRF-dependent luciferase reporter by the indicated constructs as measured under serum free condition, normalized to the mock transfected control cells. **(B)** Subcellular localisation of MRTF-A Δ3-5 by immunofluorescence microscopy of transiently transfected NIH3T3 fibroblasts using anti-HA staining. **(C)** Immunoblotting of ectopically expressed HA-tagged MRTF-A proteins. HCT-8 cells were transiently transfected with the indicated constitutive expression plasmids, or stably transfected with the floxed targeting vector depicted in Figure 1. Following stable selection by G418, cells were treated with recombinant HTNC-Cre recombinase or left untreated, as indicated. (**D**) Activation of the MRTF/SRF-dependent luciferase reporter by the indicated constructs as measured under serum free condition, normalized to the mock transfected or untreated control HCT-8 cells. Error bars, SEM (n=3). $**p \leq 0.01$, $***p \leq 0.001$. Scale bars, 20 µm.

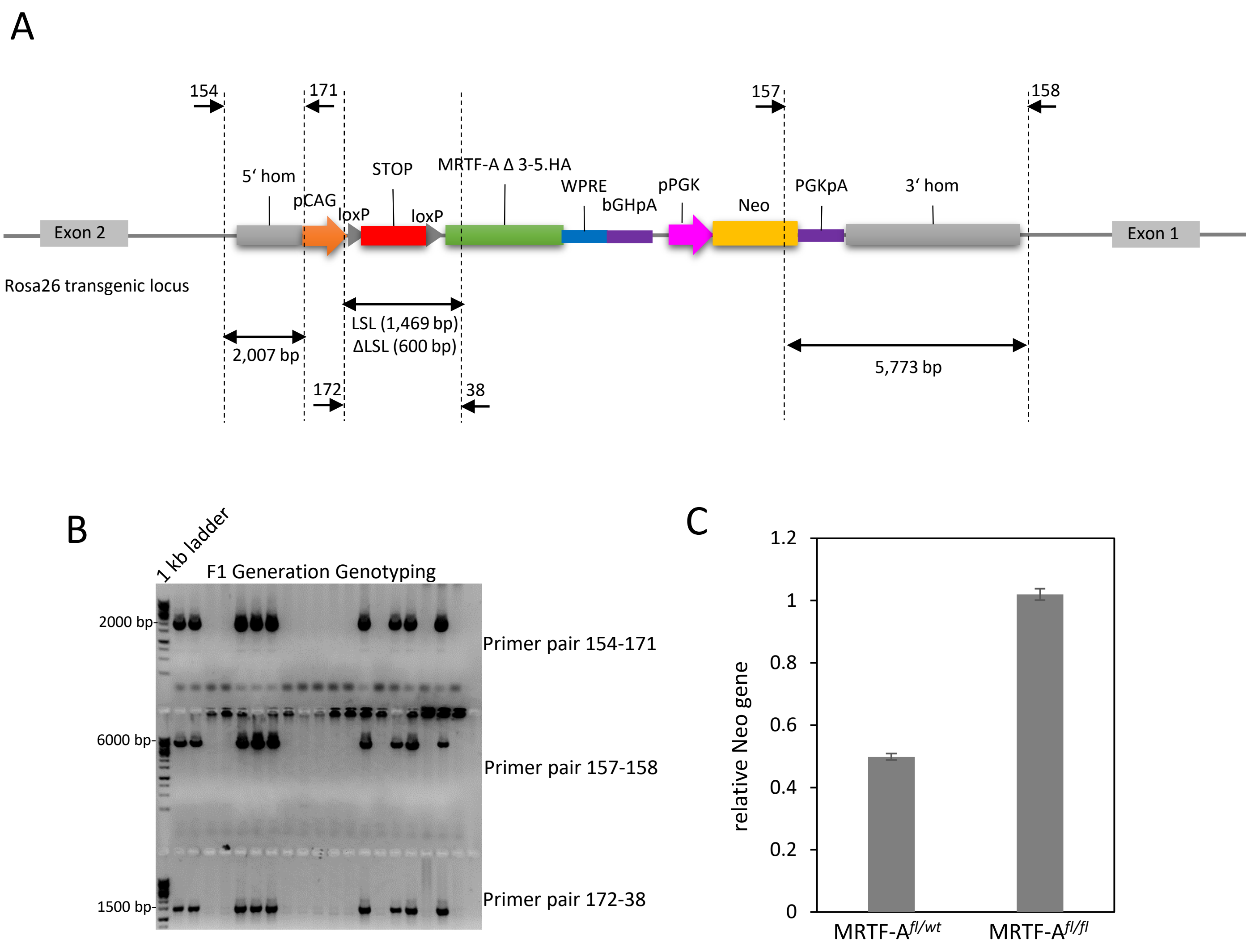

Supplementary Figure S2: Generation of a transgenic mouse line with a conditional MRTF-A gain-of-function allele. (A) Schematic representation of the transgene after homologous recombination with the ROSA26 locus. The dashed lines show the primer pairs for validation of the ES cells with homologous recombination and were subsequently used for genotyping of the generated mice. Primer pair 38 and 172 were also used to determine the presence (LSL) or the absence (ΔLSL) of the STOP cassette after Cre-induced recombination. **(B)** Genotyping of the F1 generation of the transgenic mice. **(C)** Quantitative PCR of the Neo gene on the genomic DNA isolated from the hetero- or homozygous mice, normalised to GAPDH.

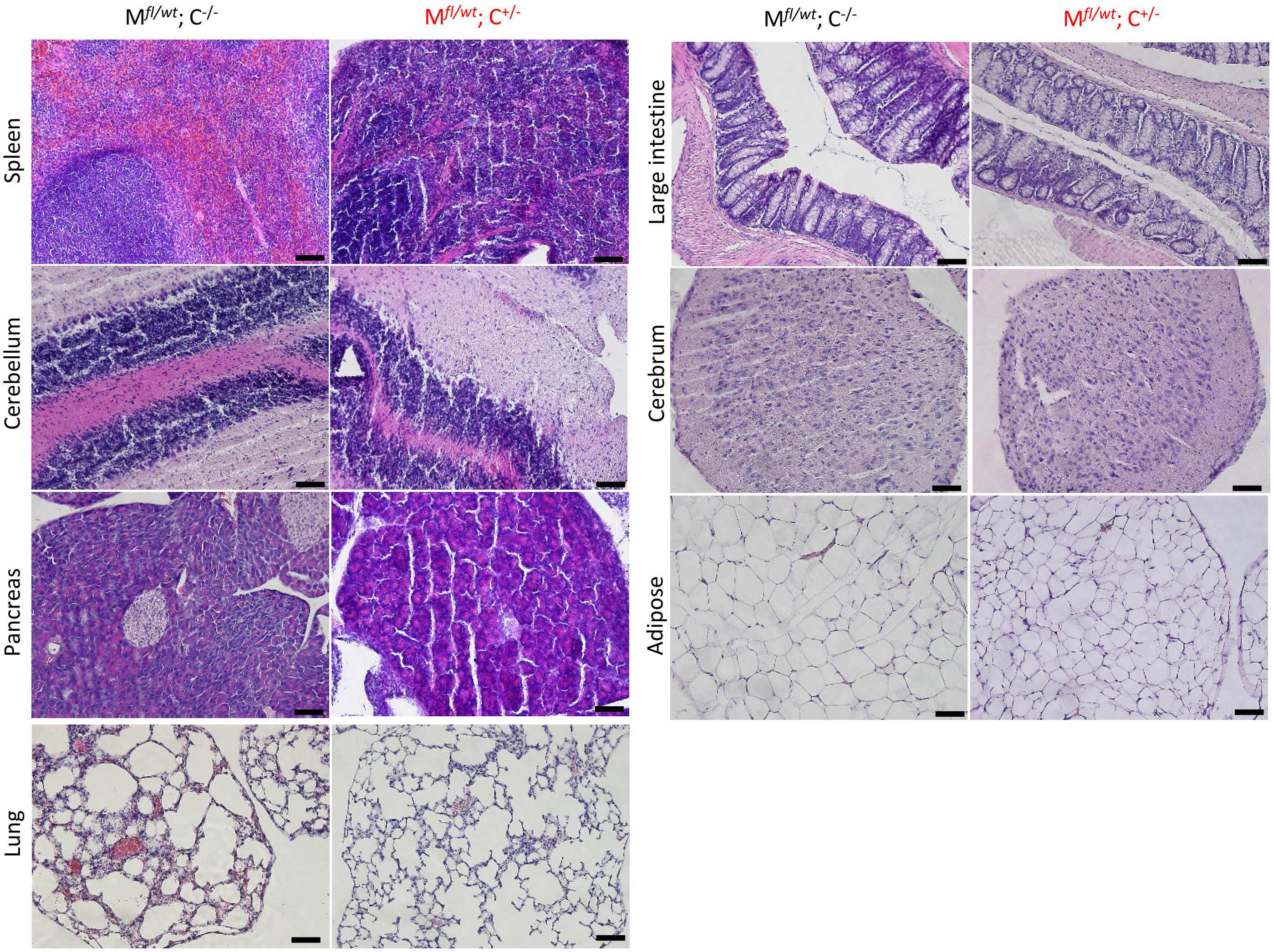

Supplementary Figure S3: Organ analysis in adult mice with induced ubiquitous expression of MRTF-A Δ3-5. Double positive mice ($M^{fl/wt}$ $C^{+/-}$) and control mice ($M^{fl/wt}$ $C^{-/-}$) were injected with 1 mg of tamoxifen on three consecutive days and analyzed one day later. Shown are H&E stainings of the indicated organs. See Figure 1 for details. Scale bars, 100 µm.

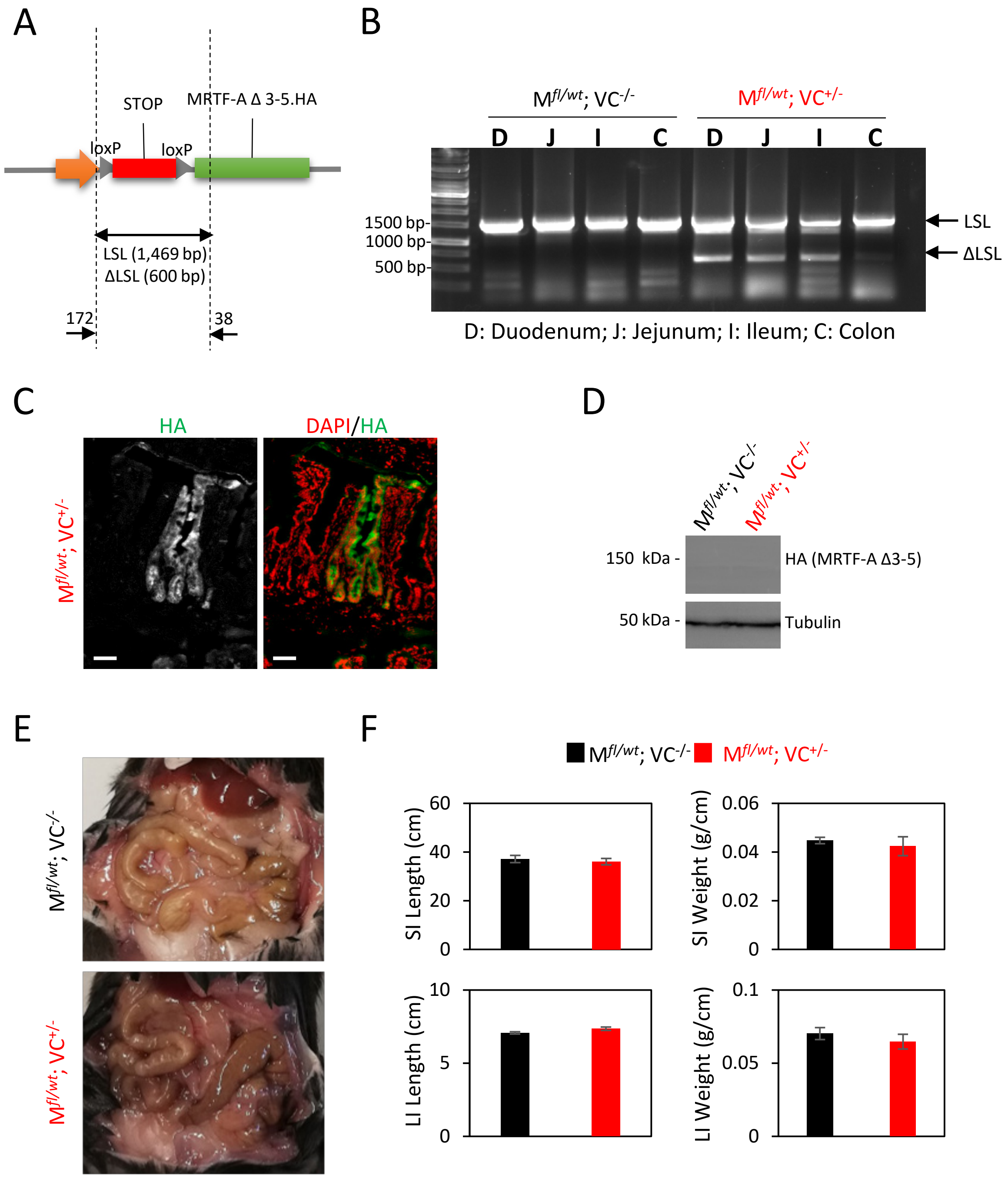

Supplementary Figure S4: Genotype and phenotype at day 60 after tamoxifen-induced activation of MRTF-A in the gut. Double positive mice ($M^{fl/wt}$ $VC^{+/-}$) and control mice ($M^{fl/wt}$ $VC^{-/-}$) were injected once with 1 mg of tamoxifen and analyzed 60 days later (see Figure 2). **(A)** Scheme showing part of the transgene relevant for recombination, and the amplicons before and after Villin-CreERT2 induction. **(B)** Genotype of indicated intestinal segments by PCR 60 days after tamoxifen. **(C)** Immunofluorescence staining of a selected area of the small intestine, showing a remaining HA-positive crypt-villus structure. **(D)** Immunoblotting of intestinal protein extracts with anti-HA antibodies and tubulin as a control. **(E)** Macroscopic picture of the mice after 60 days. **(F)** Length and relative weight of the small intestine (SI) and the large intestine (LI) after 60 days. Error bars, SEM (n=3). Scale bars, 50 µm.

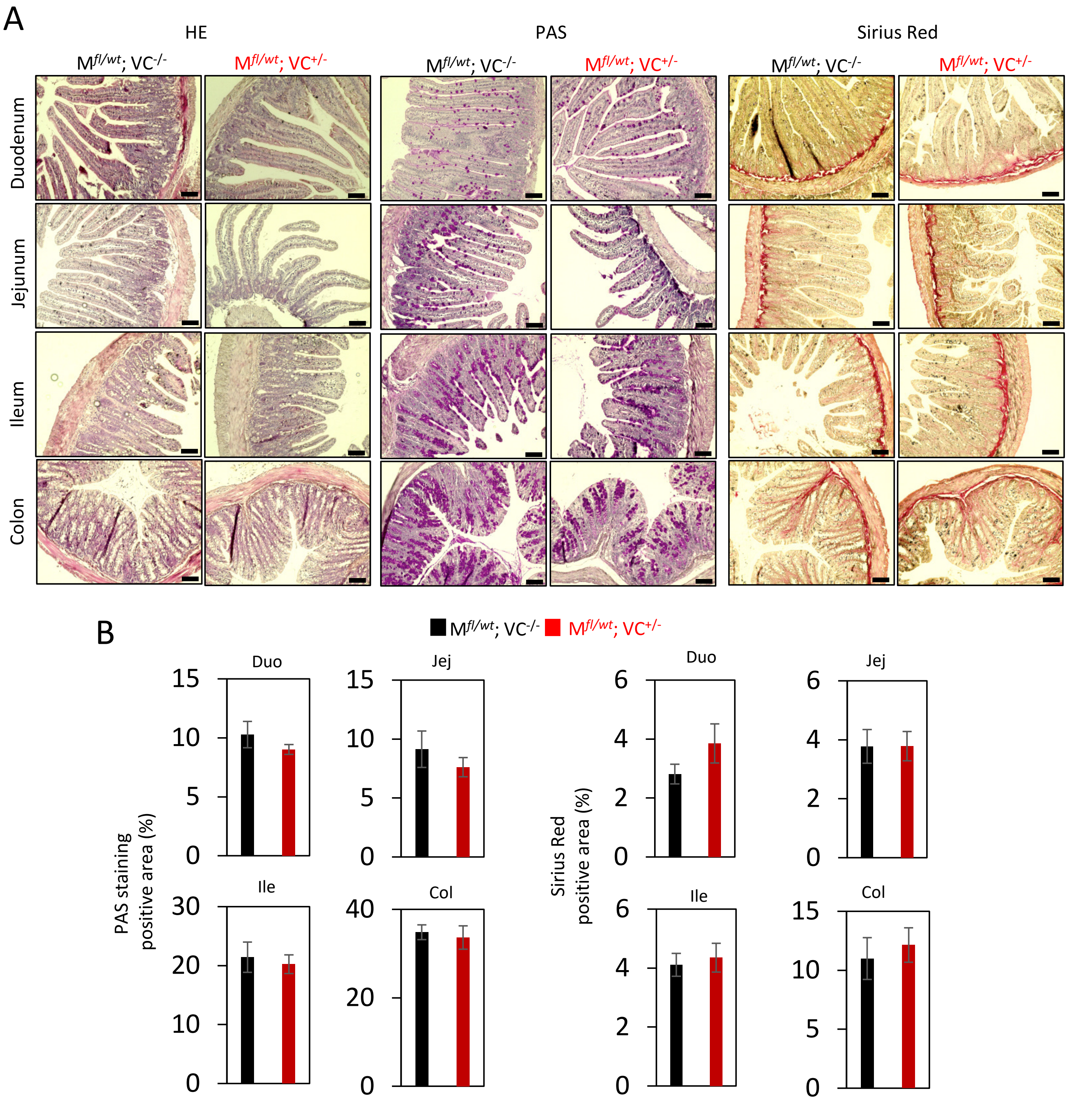

Supplementary Figure S5: Intestinal histology at day 60 after tamoxifen-induced activation of MRTF-A in the gut. Double positive mice ($M^{fl/wt}$ $VC^{+/-}$) and control mice ($M^{fl/wt}$ $VC^{-/-}$) were injected once with 1 mg of tamoxifen and analyzed 60 days later (see Figure 4). **(A)** Histological analysis of distinct intestinal regions by H&E staining, PAS staining for mucins, and Sirius Red staining for collagen-rich connective tissue. **(B)** Quantification of the areas positive for PAS- and Sirius Red staining. Scale bars, 50 µm.

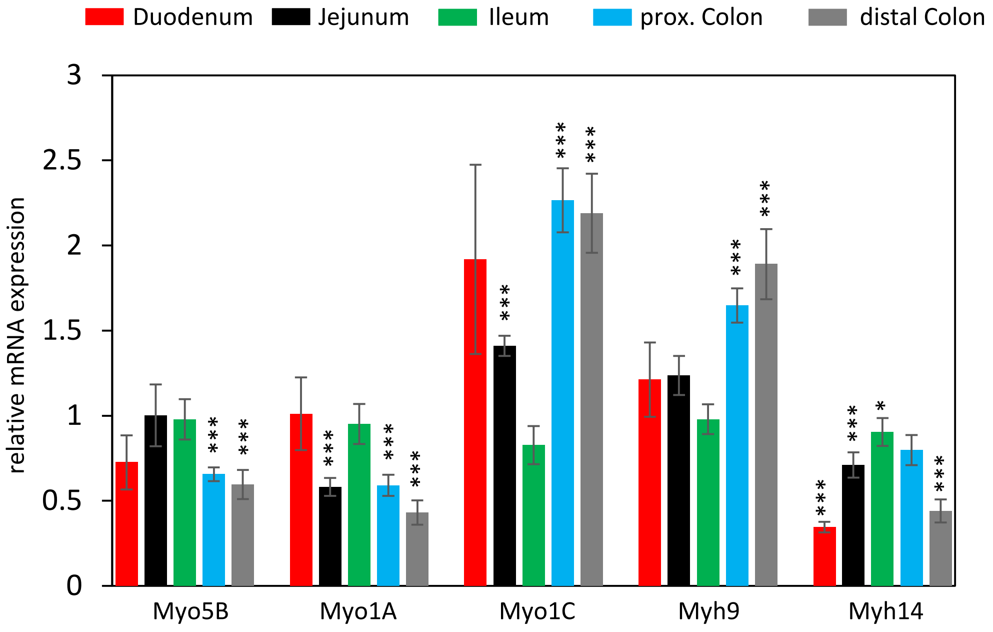

Supplementary Figure S6: Myosin gene expression upon MRTF-A activation in the murine gut at day 7. Double positive mice and control mice were injected once with 1 mg of tamoxifen and RNA was prepared 7 days later from the five indicated intestinal regions (see Figure 8). Bar colors represent the duodenum, jejunum, ileum, proximal colon and distal colon, as indicated. Relative mRNA of indicated myosins in comparison to the single-positive control mice. Error bars, SEM (n=3). *$p \leq 0.05$, **$p \leq 0.01$, ***$p \leq 0.001$.

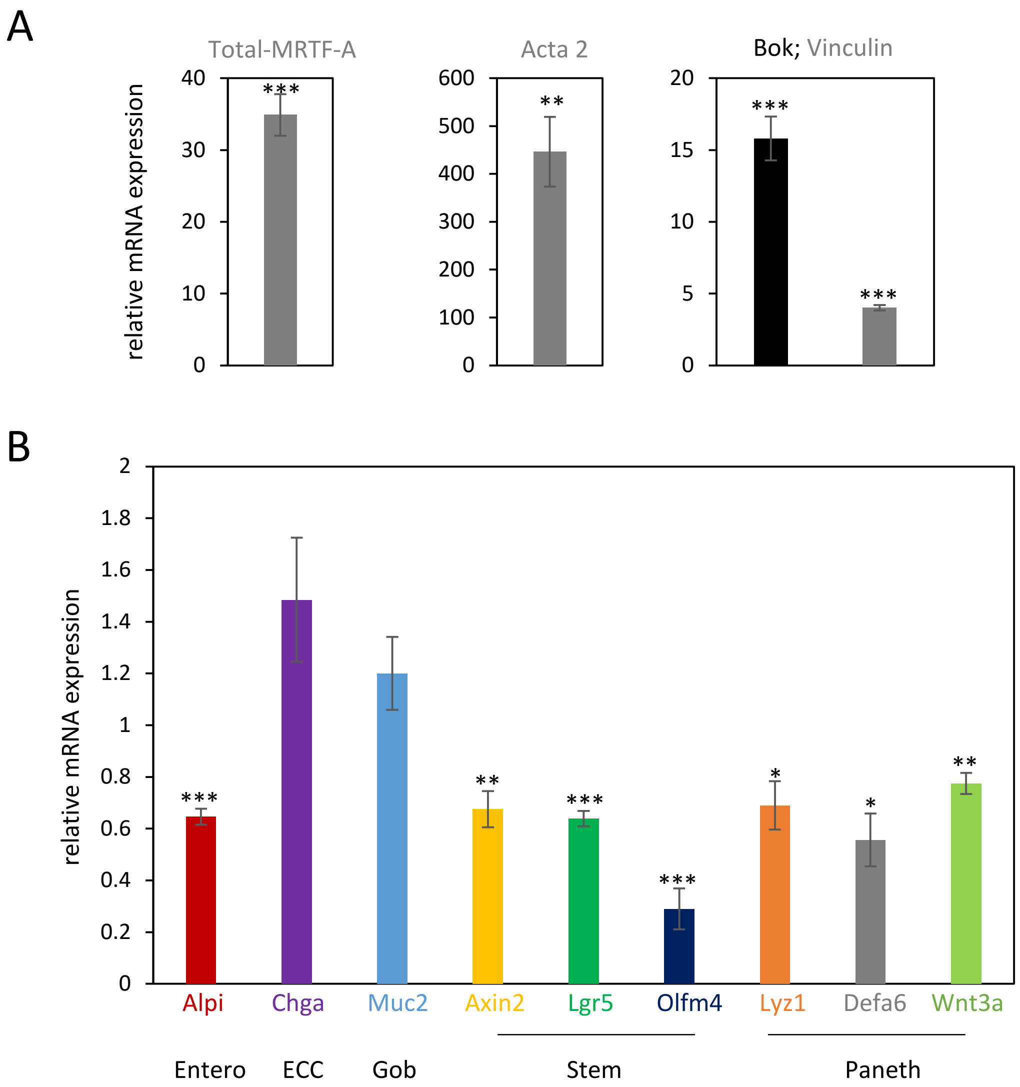

Supplementary Figure S7: Relative changes in mRNA expression in intestinal organoids upon MRTF-A gain of function. RNA was prepared from the control and double positive organoids after two days of tamoxifen treatment. qRT-PCR was performed with intron-spanning primer pairs for the genes indicated, and normalized to housekeeping control genes. **(A)** Relative mRNA of MRTF-A transcripts (including the transgenic) in comparison to the single-positive control mice and MRTF-A target genes Acta2, Bok and Vlc. **(B)** Marker genes for intestinal cell types, as indicated underneath. Entero, enterocytes; ECC, enterochromaffine cells; Gob, goblet cells; Stem, stem cells; Paneth, paneth cells. Error bars, SEM (n=3). *p ≤ 0.05, **p ≤ 0.01, ***p ≤ 0.001.

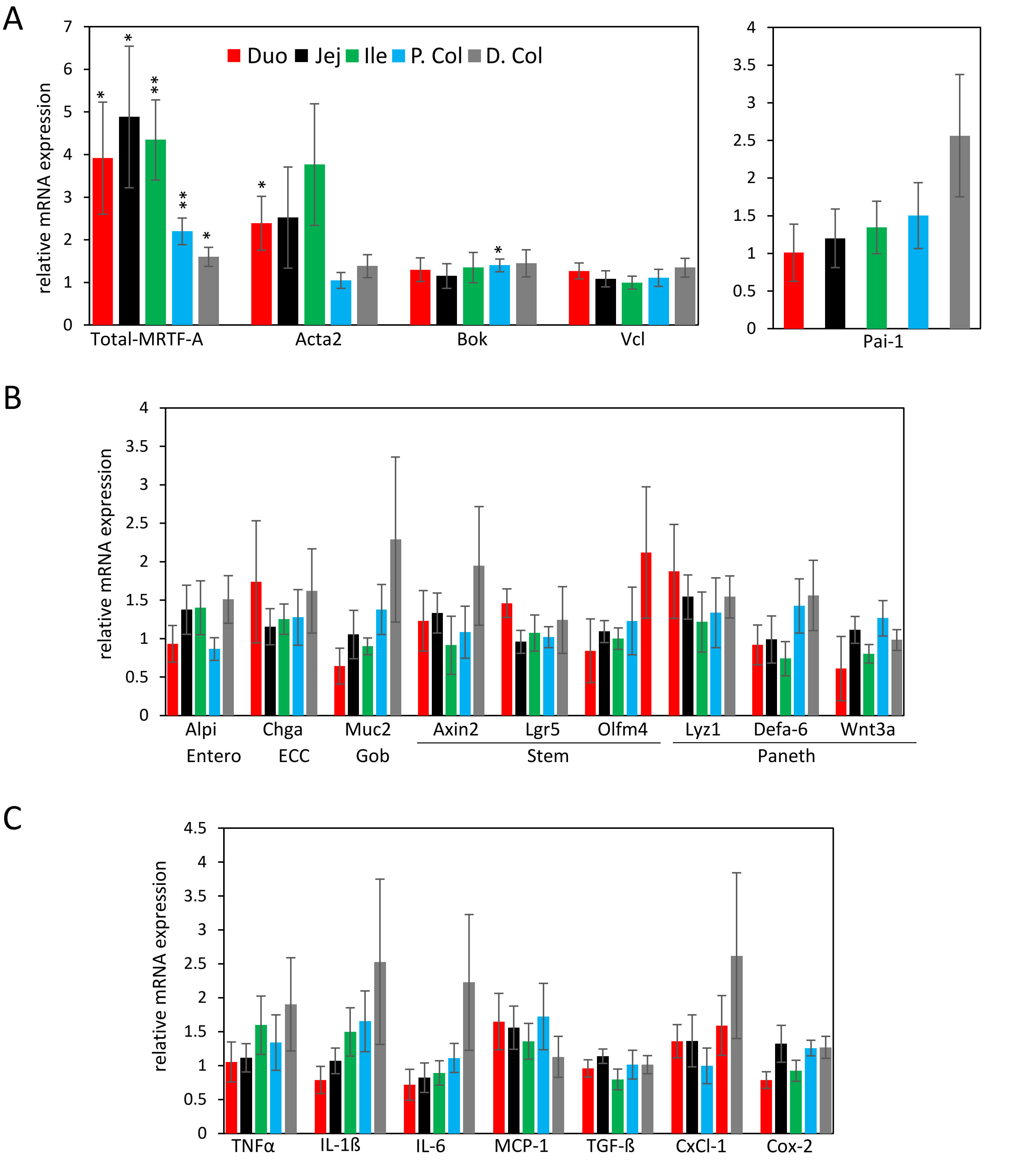

Supplementary Figure S8: Long-term gene expression changes upon MRTF-A activation in the gut. Double positive mice and control mice were injected once with 1 mg of tamoxifen and RNA was prepared 60 days later from the five indicated intestinal regions (see Figure 8). Bar colors represent the duodenum, jejunum, ileum, proximal colon and distal colon, as indicated. **(A)** Relative mRNA of MRTF-A transcripts (including the transgenic) and the MRTF-A target genes Acta2, Bok, Vcl and Pai-1 in comparison to the single-positive control mice. **(B)** Marker genes for intestinal cell types as indicated underneath. Entero, enterocytes; ECC, enterochromaffine cells; Gob, goblet cells; Stem, stem cells; Paneth, paneth cells. **(C)** Inflammatory marker genes in the intestine, as indicated. Error bars, SEM (n=3). *$p \leq 0.05$, **$p \leq 0.01$, ***$p \leq 0.001$.

Supplementary Table S1: Oligonucleotides used in this study

Primer	Sequence 5' -> 3'	Description
154	CGTGTGGTTCGGTGTCTCTT	5' homology arm detection
171	TAGGGGGCGTACTTGGCATA	
172	GGCAACGTGCTGGTTATTGT	Detction of STOP cassette
38	GGACTCCACAGGCAGGATATT	
157	AGTGTTGAGGGCAATCTGGG	3' homology arm detection
158	CAAGATGGATTGCACGCAGG	
floxed-f	GGCAACGTGCTGGTTATTGT	Genotyping
floxed-r	GGACTCCACAGGCAGGATATT	
Cre-f	AACATGCTTCATCGTCGG	Genotyping
Cre-r	TTCGGATCATCAGCTACACC	
Gapdh-f	GGTATCGTGGAAGGACTCATG	qPCR
Gapdh-r	ATGCCAGTGAGCTTCCCGTTC	
Hprt-f	AAGCTTGCTGGTGAAAAGGA	qPCR
Hprt-r	TTGCGCTCATCTTAGGCTTT	
Axin2-f	GGACTGGGGAGCCTAAAGGT	qPCR
Axin2-r	AAGGAGGGACTCCATCTACGC	
Lgr5-f	CTGAGACAGGTTCCGGAGGA	qPCR
Lgr5-r	GAGATGCAGAACCACGAGGC	
Olfm4-f	GCCACTTTCCAATTTCAC	qPCR
Olfm4-r	GAGCCTCTTCTCATACAC	
Lyz1-f	GGAATGGATGGCTACCGTGG	qPCR
Lyz1-r	CATGCCACCCATGCTCGAAT	
Defa6-f	CCTTCCAGGTCCAGGCTGAT	qPCR
Defa6-r	TGAGAAGTGGTCATCAGGCAC	
Wnt3-f	TGGAACTGTACCACCATAGATGAC	qPCR
Wnt3-r	ACACCAGCCGAGGCGATG	
Alpi-f	GGCCATCTAGGACCGGAGA	qPCR
Alpi-r	TGTCCACGTTGTATGTCTTGG	
Chga-f	CCAAGGTGATGAAGTGCGTC	qPCR
Chga-r	GGTGTCGCAGGATAGAGAGGA	
Muc2	ATGCCCACCTCCTCAAAGAC	qPCR
Muc2-r	GTAGTTTCCGTTGGAACAGTGAA	
Mrtf-a-f	GAGAAGAATATCCTGCCTGTG	qPCR
Mrtf-a-r	GAGTTGAGAAAGAACCTGAGTG	
Acta2-f	GTCCCAGACATCAGGGAGTAA	qPCR
Acta2-r	TCGGATACTTCAGCGTCAGGA	
Bok-f	GGCAAGGTAGTGTCCCTGTA	qPCR
Bok-r	GCTCATCTCTCTGGCAACAAC	
Vlc-f	GGCCGGACCAACATCAGTG	qPCR
Vlc-r	ATGTACCAGCCAGATTTGACG	
TNF-a-f	TCGTAGCAAACCACCAAGTG	qPCR
TNF-a-r	TTGTCTTTGAGATCCATGCC	
IL1-b-f	GCTTCCTTGTGCAAGTGTCT	qPCR
IL1-b-r	GGTGGCATTTCACAGTTGAG	
IL6-f	AGTCCGGAGAGGAGACTTCA	qPCR
IL6-r	TTGCCATTGCACAACTCTTT	
MCP1-f	GCAGCAGGTGTCCCAAAGAA	qPCR
MCP1-r	ATTTACGGGTCAACTTCACATTCAA	
TGF-b-f	ACTGATACGCCTGAGTGGCT	qPCR
TGF-b-r	CCCTGTATTCCGTCTCCTTG	
Cxcl1-f	CCGAAGTCATAGCCACACTC	qPCR
Cxcl1-r	GTGCCATCAGAGCAGTCTGT	
COX2-f	CAGACAACATAAACTGCGCCTT	qPCR
COX2-r	GATACACCTCTCCACCAATGACC	
CFTR-f	TTCTTCACGCCCCTATGTCGA	qPCR
CFTR-r	GCTCCAATCACAATGAACACCA	
Pai-1-f	AGTGGACTTTTCAGAGGTGGA	qPCR
Pai-1-r	GCCGTTGAAGTAGAGGGCATT	
Myo5B-f	AACCAAATCCTGTGCCAGTCA	qPCR
Myo5B-r	CCCGGAGTTTGCTCGTCCC	
Myo1A-f	GCGGTGTCATCACCAACTATCT	qPCR
Myo1A-r	TCCCCATTCAGGTAGCCGTA	
Myo1C-f	GAGCAACCCCGTGTTAGAGG	qPCR
Myo1C-r	ACTTTTCCAGGAGGTAACTGAGA	
Myh9-f	AGAACATGGACCCCTTGAACG	qPCR
Myh9-r	TGATCCGATCCACATCCTTCC	
Myh14-f	GTCAGCACCATGTCTTATGGG	qPCR
Myh14-r	TTTGCCAAATCGGGAAGAGTT	
