# Supplementary material for: MRTF-A gain-of-function in mice impairs homeostatic renewal of the intestinal epithelium
Source: Cell Death Dis. 2023 Sep 28;14(9):639. doi: 10.1038/s41419-023-06158-4 (PMC10539384; doi:10.1038/s41419-023-06158-4)
Supplement: Supplementary file 2 — Fullsize uncropped western blots [file 41419_2023_6158_MOESM2_ESM.pdf]

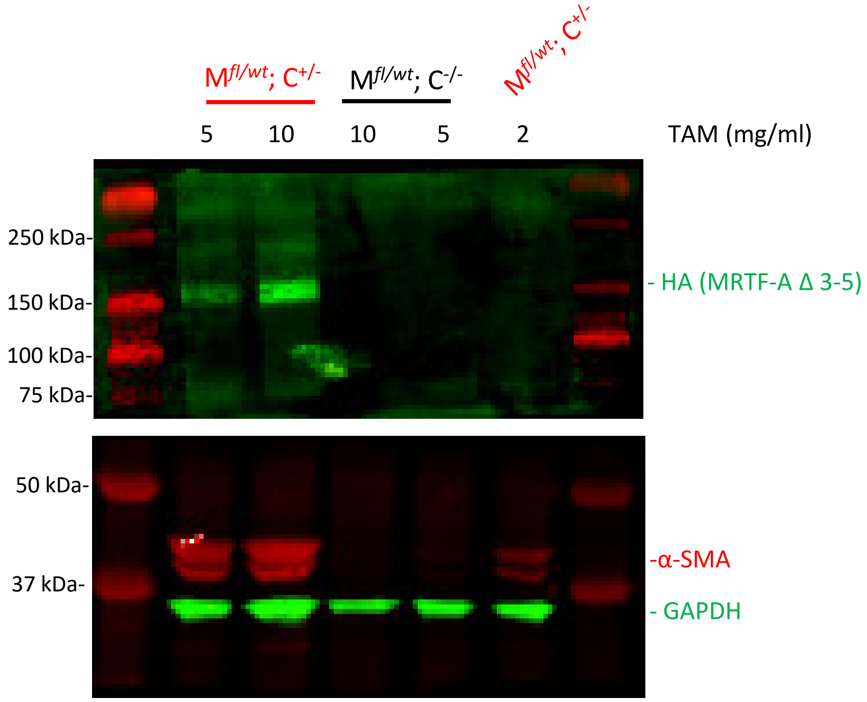

Fullsize blots Figure 3B

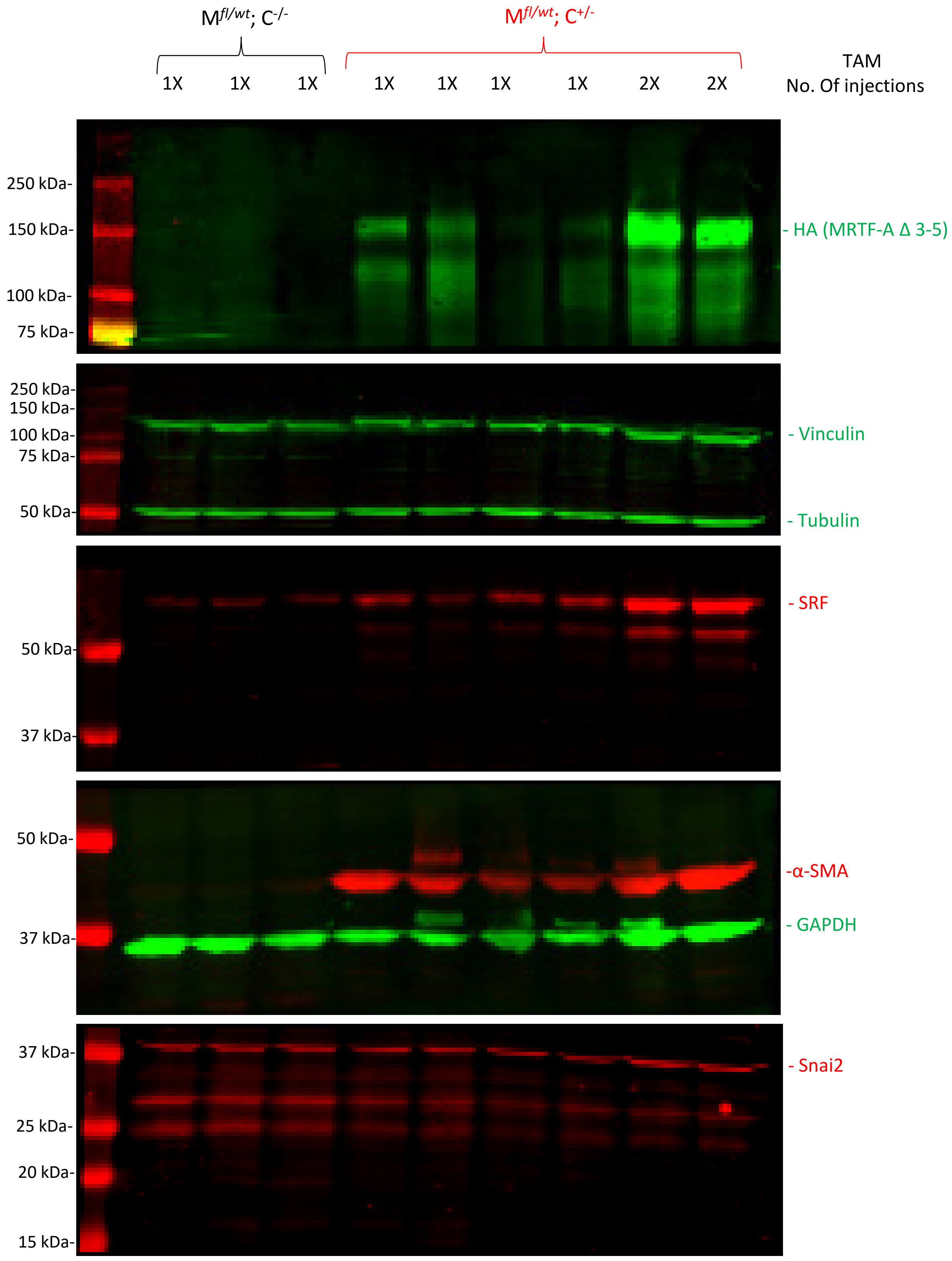

Fullsize blots Figure 3C

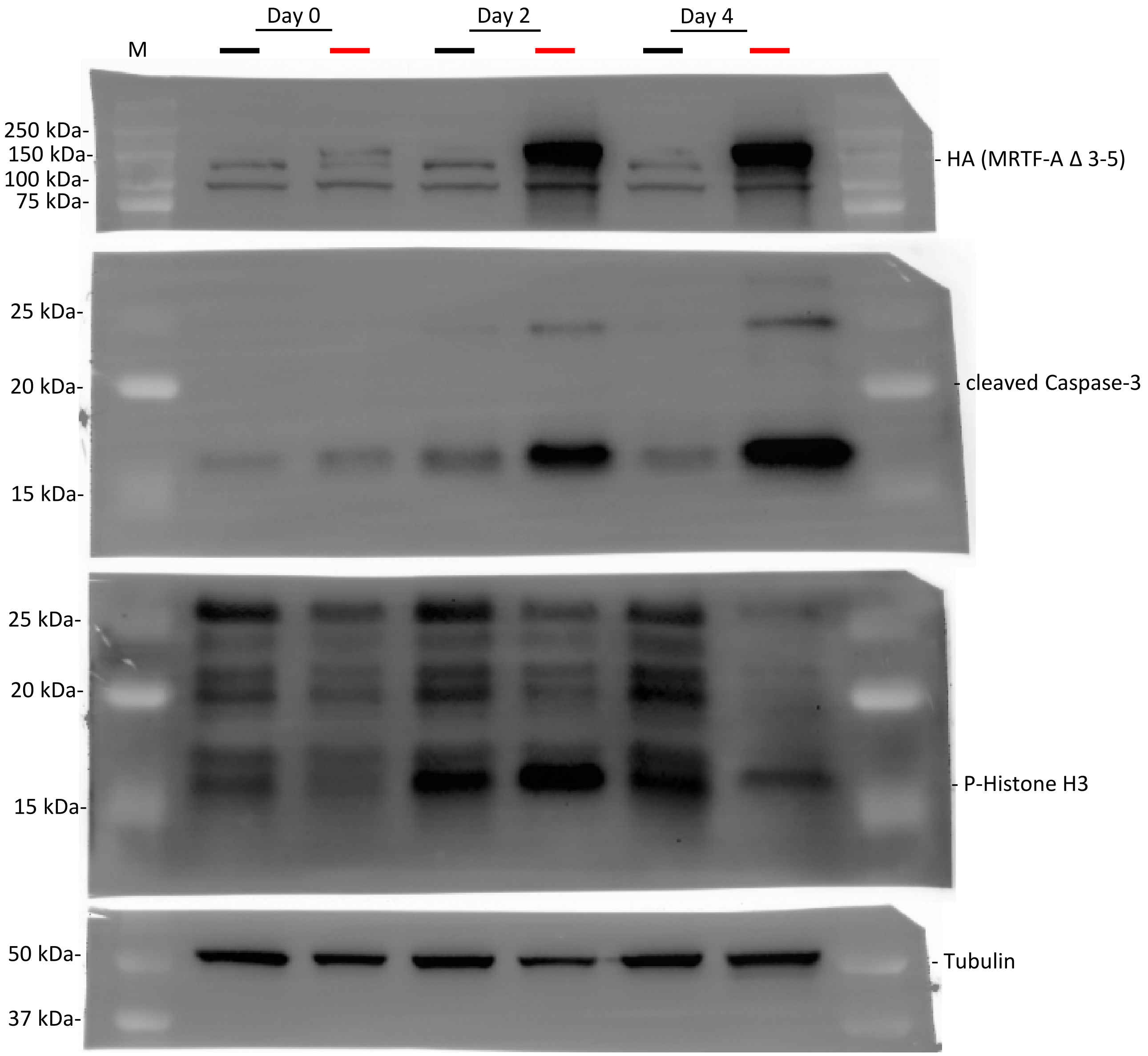

Fullsize blots Figure 6

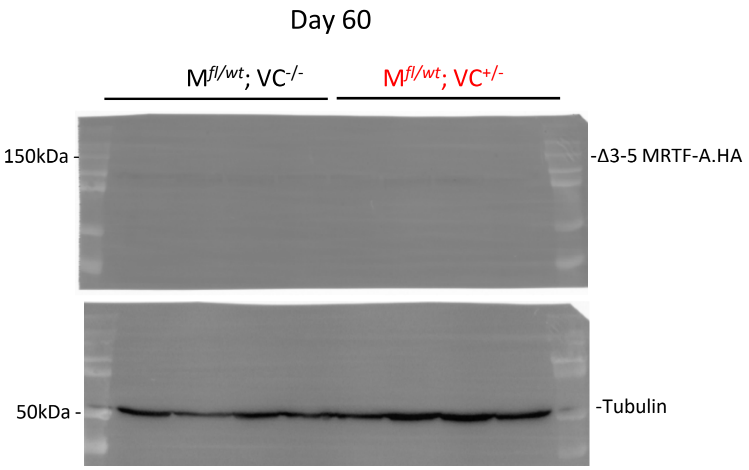

Fullsize blots Suppl. Figure S4D
